# Subtype assignment of CLL based on B-cell subset associated gene signatures from normal bone marrow – A proof of concept study

**DOI:** 10.1371/journal.pone.0193249

**Published:** 2018-03-07

**Authors:** Caroline Holm Nørgaard, Lasse Hjort Jakobsen, Andrew J. Gentles, Karen Dybkær, Tarec Christoffer El-Galaly, Julie Støve Bødker, Alexander Schmitz, Preben Johansen, Tobias Herold, Karsten Spiekermann, Jennifer R. Brown, Josephine L. Klitgaard, Hans Erik Johnsen, Martin Bøgsted

**Affiliations:** 1 Department of Haematology, Aalborg University Hospital, Aalborg, Denmark; 2 Department of Clinical Medicine, Aalborg University, Aalborg, Denmark; 3 Departments of Medicine and Biomedical Data Science, Stanford, California, United States of America; 4 Clinical Cancer Research Center, Aalborg University Hospital, Aalborg, Denmark; 5 Department of Pathology, Aalborg University Hospital, Aalborg, Denmark; 6 Department of Internal Medicine 3, University of Munich, Munich, Germany; 7 Department of Medical Oncology, Dana-Farber Cancer Institute, Boston, MA, United States of America; 8 Department of Medicine, Brigham and Women's Hospital, Harvard Medical School, Boston, MA, United States of America; University of Texas MD Anderson Cancer Center, UNITED STATES

## Abstract

Diagnostic and prognostic evaluation of chronic lymphocytic leukemia (CLL) involves blood cell counts, immunophenotyping, IgVH mutation status, and cytogenetic analyses. We generated B-cell associated gene-signatures (BAGS) based on six naturally occurring B-cell subsets within normal bone marrow. Our hypothesis is that by segregating CLL according to BAGS, we can identify subtypes with prognostic implications in support of pathogenetic value of BAGS. Microarray-based gene-expression samples from eight independent CLL cohorts (1,024 untreated patients) were BAGS-stratified into pre-BI, pre-BII, immature, naïve, memory, or plasma cell subtypes; the majority falling within the memory (24.5–45.8%) or naïve (14.5–32.3%) categories. For a subset of CLL patients (*n* = 296), time to treatment (TTT) was shorter amongst early differentiation subtypes (pre-BI/pre-BII/immature) compared to late subtypes (memory/plasma cell, HR: 0.53 [0.35–0.78]). Particularly, pre-BII subtype patients had the shortest TTT among all subtypes. Correlates derived for BAGS subtype and IgVH mutation (*n* = 405) revealed an elevated mutation frequency in late vs. early subtypes (71% vs. 45%, *P* < .001). Predictions for BAGS subtype resistance towards rituximab and cyclophosphamide varied for rituximab, whereas all subtypes were sensitive to cyclophosphamide. This study supports our hypothesis that BAGS-subtyping may be of tangible prognostic and pathogenetic value for CLL patients.

## Introduction

Patients with chronic lymphocytic leukemia (CLL) experience a variable disease course. Some demonstrate slow progression and survive for decades, while others rapidly succumb to chemotherapy-resistant disease [1]. The prognostic assessment of CLL patients at diagnosis ordinarily employs the Rai [2] or Binet [3] clinical staging systems, together with assessments of chromosomal and IgVH mutation status and, more recently, *TP53*, ZAP-70, and CD38 evaluations. Risk scores implementing current available markers have been developed [4–5], while advances in genomic technologies have facilitated new tools to address prognosis [6–8], and revealing substantial genetic and epigenetic heterogeneity in CLL [9].

The precise cell-of-origin in CLL remains under debate [10–11] and any direct link to a normal B-cell subset has proven difficult given that no single B-cell population shares the unique CD5^+^, CD19^+^, CD20^+^, and CD23^+^ immunophenotype characteristic of CLL [6]. With this in mind, we sought to identify differentiation patterns expressed in end-stage CLL cells, using these to classify the disease into subtypes that resemble normal bone marrow (BM) B-cell subsets. We anticipated that this more sophisticated segregation of CLL could be of prognostic value, and would contribute to our understanding of CLL pathogenesis. As the first step in achieving this aim, we recently generated B-cell associated gene signatures (BAGS) for the different developmental stages of normal B-cells in blood, tonsils, thymus, and BM [12]. These BAGS signatures serve as a reference material against which tumor derived samples can be challenged. BAGS assignments can be made for clinical lymphoid and leukemic tumor samples [12–14] by statistical modeling. This enables us to classify B-cell malignancies in terms of their cellular phenotype, which may, in turn, generate insights into clonal selection and evolution.

Proof of this concept is supported by our identification of BAGS subtypes of prognostic relevance in diffuse large B-cell lymphoma [14] and multiple myeloma [15]^.^ In the present study, we BAGS-categorized individual CLL patients in order to derive correlates for prognosis, and determine the pathogenetic value of this novel classification system in CLL.

## Methods

### Collection and processing of normal tissue

Prior to study commencement, the Health Research Ethics Committee for the North Denmark Region approved our study protocol (MSCNET, N-20080062MCH). Following informed written consent, obtained in accordance with the Declaration of Helsinki, normal BM was collected from the sternum of seven adult patients undergoing cardiac surgery as described in the S1 Appendix, and elsewhere [12]. Fluorescence-activated cell sorting (FACS) was used to fractionate mononuclear BM cells into six distinct normal B-cell subsets: pre-BI (BI), pre-BII (BII), immature (IM), naïve (NA), memory (ME), and plasma cells (PC) (S1 Appendix and S1 Table). For gene expression profiling (GEP), mRNA from B-cell subsets were isolated and hybridized to the Human Exon 1.0 ST (Exon) array platform [12]^.^ A total of 38 CEL files containing B-cell data were generated using the Affymetrix Command Console. The CEL files and metadata were adjusted for compliance (with the requirements of *Minimum Information About a Microarray Experiment*) [16], and then deposited in the NCBI Gene Expression Omnibus repository (accession code GSE68878). In the present study, these data are referred to as the sternal BM B-cell data.

#### CLL data sets and study variables

We queried the NCBI Gene Expression Omnibus repository (see S1 Appendix) for microarray-based gene expression CLL data collected using the Exon and Affymetrix Human Genome U133 plus 2.0 (HG-U133plus2) arrays [17]. This led to the identification of eight CLL cohorts that included 1,024 untreated patients (S2 Table) from whom appropriate consent permissions were obtained prior to data deposits. Clinical data of varying degrees of completeness were available (S3 Table).

In brief, centers providing clinical cohorts were focused on the use of GEP data in exploring new prognostic factors [18–19], scores [20], subnetworks [21], gene signatures [22], leukemia classifications [23], pathway analysis [24], therapeutic predictions [22], and lastly, a clinical drug trial [25].

Data for time from diagnosis to the initiation of treatment (TTT) were available and combined for 296 patients in the Munich, IIDFCI (Dana Farber Cancer Institute, cohort II), and UCSD cohorts (S3 Table), which allowed us to examine CLL followed by the watch-and-wait approach. Data for overall survival were available for 108 patients in the Munich cohort, although any analyses of these data were precluded by a lack of events within the relatively short follow-up period.

#### BAGS and REGS classification

Sternal BM B-cell data were normalized using the robust multichip average (RMA) [26] method available in the *affy* package in Bioconductor, with a custom Chip Description File (CDF) used to remap probes into sets corresponding to Ensembl gene IDs (Ensembl release 81) [17].

The BAGS classifier was generated from the sternal BM B-cell data by regularized multinomial regression [27] using subtype as the response and median centered gene expression as explanatory variables. Regularization was performed by elastic net [28] where the optimal regularization parameters (alpha and lambda) were selected by cross-validation. To avoid patient-specific signatures in the classifier, the cross-validation folds were patient-specific, containing 4 or 6 samples each. To enable use on other microarray platforms, only genes available on HG-U133plus2, Affymetrix Human Genome U133 A, and Exon arrays were considered. To compensate for cohort-wise technical batch effects and array differences when classifying the patients, each clinical cohort was median centered and adjusted gene–wise to have the same variance as the sternal BM B-cell data set. To validate the compatibility of the Exon array-based classifier on other platforms, we BAGS-classified a validation data set comprising previously published (sorted) healthy B-cell subsets using the HG-U133plus2 array [12].

Post validation, CLL samples were classified according to their highest predicted probability of a subtype match, while allowing 15% of samples (with the minimum probability threshold for classification) within each cohort to remain unclassified.

Previously, resistance gene signatures (REGS) that predict the probability of resistance towards rituximab (R) and cyclophosphamide (C) have been established following the same approach as for the BAGS classifier, as described elsewhere [29–30]. REGS were generated by combining in vitro drug screening and global gene expression analyses using a panel of human B-cell cancer cell lines. To further characterize BAGS subtype properties, we estimated *expected drug resistance* based on global gene expression profiles. As a reference for malignant samples, we also assessed the inherent resistance levels predicted for normal B-cell subsets.

#### Statistical analyses

Prognostic evaluation of smoldering “watch-and-wait” CLL was performed by time to treatment (TTT), which denotes the time period from diagnosis to the initiation of treatment or censoring. Cumulative incidences were computed for TTT and differences between BAGS subtypes were tested in univariate and multivariate Cox proportional hazard regression models. Cox models for which the complete case analysis included multiple studies were adjusted for study effects, since differences in TTT between study cohorts were observed, as illustrated in S1 Fig. Fisher’s exact test was used to test for differences in the distribution of BAGS subtypes between the study cohorts, as well as between patients with mutated IgVH (mIgVH) versus (vs.) unmutated IgVH (uIgVH). To increase statistical power and emphasize trends in the TTT and IgVH analyses, the analyses were repeated after dividing the BAGS classifications into early (BI, BII, IM), NA, and late (ME, PC) subtype groups, based on the normal pre- and postgerminal B-cell differentiation hierarchy. For drug resistance determined by the REGS classifier, an F-test was used to test for equal resistance probabilities across subtypes. The significance level was set to 0.05 and effect estimates were provided with 95% confidence intervals. All statistical analyses were performed using R version 3.3.2 [31].

## Results

### Generation of the BAGS classifier

**Fig 1:**
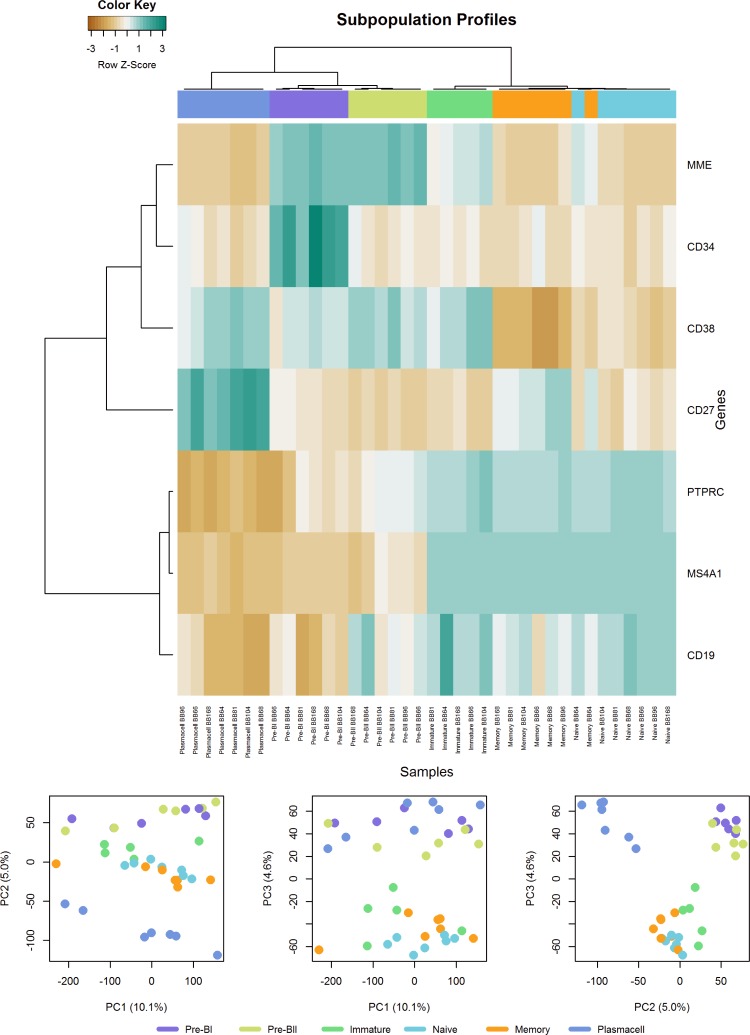
Quality assessment of the normal B-cell subsets. **a**) Unsupervised clustering of surface marker genes in the six normal B-cell subsets. Heat map showing unsupervised hierarchical clustering of normal B-cell subsets based on their expression of the cell surface markers used for FACS. The color scale indicates relative gene expression: brown, low expression; blue, high expression. Color codes: pre-BI, purple; pre-BII, yellow; immature, green; naïve, turquoise; memory, orange; and plasma cells, blue. (**b**) Principal component analysis of the global gene expression (in total 39,115 genes) in normal B-cell subsets. 1^st^, 2^nd^, and 3^rd^ principal components are shown and plotted against each other.

The six distinct B-cell subsets from normal BM were first evaluated by hierarchical clustering of gene expression using the membrane markers used for subset acquisition and sorting. This approach resulted in clusters associated with specific B-cell subsets (Fig 1A). A principal component analysis of the global gene expression discriminated distinct B-cell subsets as illustrated in Fig 1B, which shows clustering of the BI and BII subsets. Similarly, the IM, NA, and ME subsets were found to cluster together, with the PC subset grouped separately. Hence, the B-cell subsets in question could be separated based on their overall gene expression profiles supporting their use in generating the BAGS classifier.

The BAGS classifier was created by regularized multinomial regression and included a total of 184 genes (S5 Table). Each B-cell subset showed a distinct gene expression signature comprising 27–54 genes. The signatures included 49 genes associated with specific B-cell functions, 108 genes with other biological functions, and 27 genes of unknown function. Classification of sorted samples using the array validation data set achieved 100% accuracy (S4 Table), indicating the preservation of differentiation-specific signals across microarray platforms.

### BAGS assignment of CLL samples

Each CLL patient sample was BAGS-classified to the normal B-cell subtype that it most resembled according to probability scores (S2 Fig). In each cohort, 15% of samples with the lowest probability scores remained unclassified, resulting in cohort-wise probability cut-offs ranging from 0.38–0.50.

Assignment of CLL patients to one of six BAGS subtypes (BI, BII, IM, NA, ME, and PC) resulted in frequencies of 10.0–18.2%, 0.0–6.2%, 0.0–10.5%, 14.5–32.3%, 24.5–45.8%, and 1.8–5.5%, respectively (Table 1). Despite a statistically significant difference in the distribution of BAGS subtypes across cohorts (*P* = 0.02), we consider distribution patterns to be comparable, as shown in S3 Fig.

**Table 1:** Subtype classification according to BAGS for CLL sample cohorts.

Cohort	*n*	BAGS Subtypes n (%)						
		Pre-BI	Pre-BII	Immature	Naive	Memory	Plasma cell	Unclassified
**DUKE**	68	12 (17.6)	1 (1.5)	5 (7.4)	13 (19.1)	23 (33.8)	3 (4.4)	11 (16.2)
**IDFCI**	124	15 (12.1)	4 (3.2)	13 (10.5)	21 (16.9)	49 (39.5)	3 (2.4)	19 (15.3)
**IIDFCI**	83	11 (13.3)	0 (0)	6 (7.2)	12 (14.5)	38 (45.8)	3 (3.6)	13 (15.7)
**MUNICH**	127	18 (14.2)	5 (3.9)	5 (3.9)	30 (23.6)	43 (33.9)	7 (5.5)	19 (15.0)
**PADOVA**	112	14 (12.5)	4 (3.6)	7 (6.2)	17 (15.2)	51 (45.5)	2 (1.8)	17 (15.2)
**ROCHE**	318	58 (18.2)	10 (3.1)	16 (5.0)	93 (29.2)	78 (24.5)	15 (4.7)	48 (15.1)
**SAPIENZA**	62	8 (12.9)	2 (3.2)	0 (0.0)	20 (32.3)	20 (32.3)	2 (3.2)	10 (16.1)
**UCSD**	130	13 (10.0)	8 (6.2)	8 (6.2)	33 (25.4)	42 (32.3)	6 (4.6)	20 (15.4)
**Total**^**a**^	1024	149 (14.6)	34 (3.3)	60 (5.9)	239 (23.3)	344 (33.6)	41 (4.0)	157 (15.3)
**Range**^**b**^	62–318	10.0–18.2	0.0–6.2	0.0–10.5	14.5–32.3	24.5–45.8	1.8–5.5	15.1–16.2

^a^The total number and

^b^frequency range for each subtype is listed. Tests for significantly different distributions across data sets were calculated using Fisher’s exact test (*P* = 0.02).

### Prognostic impact of the assigned BAGS subtypes

**Fig 2:**
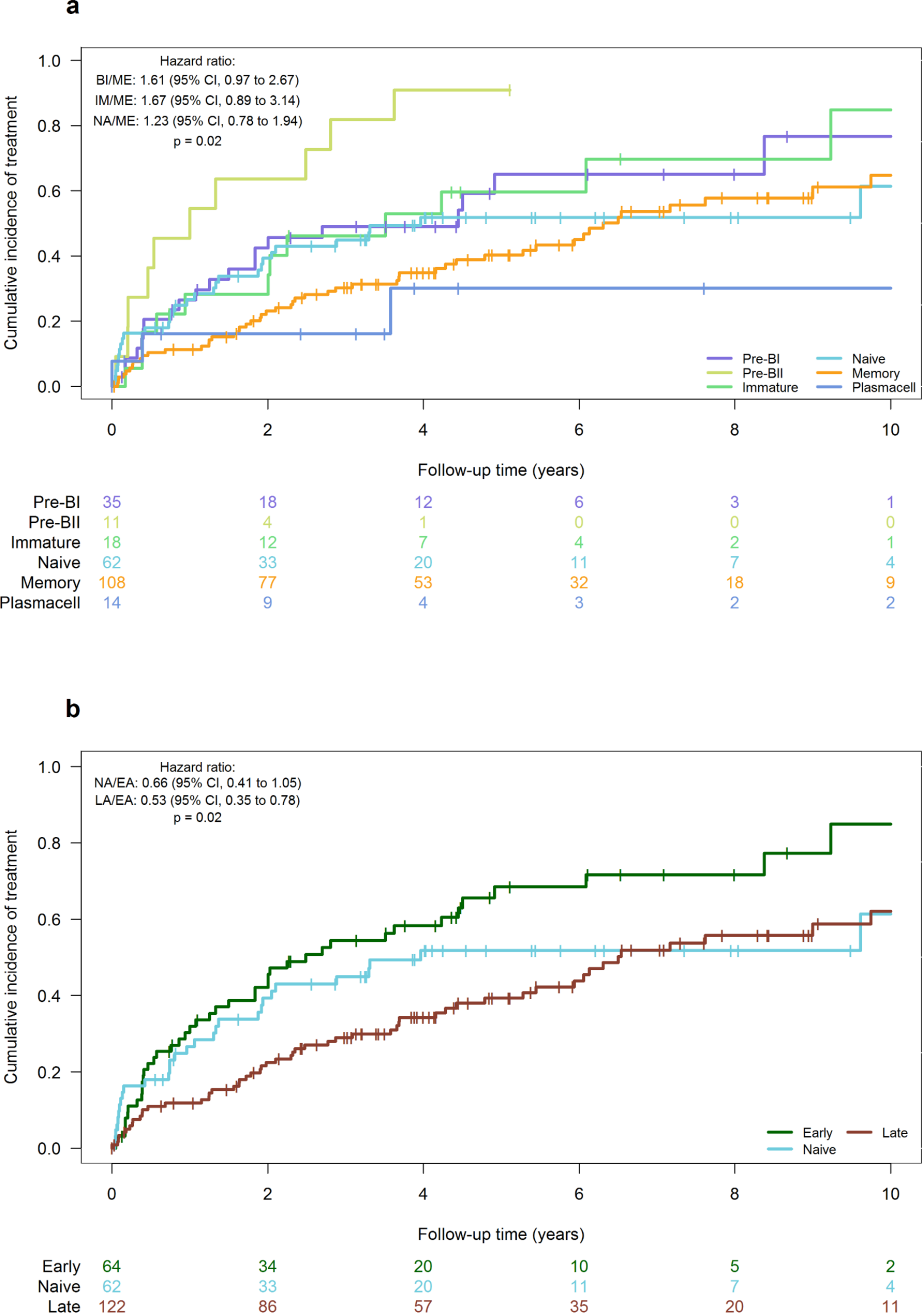
Analyses of the prognostic impact of subtyping according to BAGS on TTT in watch-and-wait CLL. Cumulative incidence curves show years elapsed from the time of diagnostic GEP until the commencement of initial treatment. (**a**) All subtypes. (**b**) All subtypes divided as early (pre-), naïve, and late (post-germinal). Color codes as in Fig 1. Data from both the Munich, IIDFCI, and UCSD cohort were used (*n* = 296).

The Munich, IIDFCI, and UCSD cohorts with available TTT data were combined (*n* = 296) and used to assess the prognostic impact of subtyping according to BAGS. The median time to the start of initial treatment was 4.91 years, with the median follow-up time calculated to be 5.45 years by the reverse Kaplan-Meier method. Patients assigned to the ME subtype had a significantly longer TTT compared to the BII (HR: 3.67[1.82–7.38], *P* < 0.001) and borderline significantly longer TTT compared to BI (HR: 1.61 [0.97–2.67]), as shown in Table 2 and Fig 2A.

**Table 2:** BAGS assignment and time to treatment.

	Hazard ratio	95% CI	*P*
**BAGS subtype**			
**Memory**	1		
**Pre-BI**	1.61	0.97 to 2.67	0.07
**Pre-BII**	3.67	1.82 to 7.38	< 0.001
**Immature**	1.67	0.89 to 3.12	0.11
**Naïve**	1.23	0.78 to 1.94	0.37
**Plasma cell**	0.80	0.32 to 2.01	0.64
**Unclassified**	1.17	0.73 to 1.90	0.51
**BAGS subtype groups**^**a**^			
**Early**	1		
**Naïve**	0.66	0.41 to 1.05	0.078
**Late**	0.53	0.35 to 0.78	0.002
**Unclassified**	0.63	0.38 to 1.03	0.067

^a^BAGS assignments for individual BAGS subtypes and grouped as early (BI, BII), NA, or late (ME, PC) were associated to outcome (time to treatment) using univariate Cox proportional hazards regression analysis. The Munich, IIDFI, and UCSD cohorts were used (*n* = 296).

In addition, by stratifying BAGS into early, NA, and late subtypes, a significantly longer TTT was observed in the late vs. early group (Table 2 and Fig 2B), with respective 2-year cumulative incidences of 23% [15%-30%] and 42% [30%-55%] (HR: 0.53[0.35–0.78], *P* = 0.002). No statistically significant difference in TTT was observed between early and NA subtypes, although the NA subgroup displayed a marginally longer TTT (HR: 0.66[0.41–1.05], *P* = 0.08), suggesting that TTT reflects the hierarchy of B-cell differentiation.

BAGS classification exhibited an independent prognostic value for TTT regarding the ME subtype compared to the BII subtype in a multivariate analysis adjusted for gender, IgVH status, and cytogenetic status (S6 Table). Furthermore, BAGS retained significance with only cytogenetic status in the model, but not with IgVH status alone.

### BAGS subtypes and clinical variables

Having identified six BAGS subtypes of prognostic relevance, we then determined whether any of these subtypes were associated with IgVH mutation status given that this is an important prognostic marker in CLL. Specifically, uIgVH is associated with an increased risk vs. patients with mIgVH [32][33]. We found that 135 (72%) and 10 (63%) of ME and PC subtypes, respectively, were mIgVH positive, while 31 (47%), 5 (38%), and 15 (43%) of BI, BII, and IM patients, respectively, were mIgVH positive (S4A Fig). Collectively, the frequency of mIgVH was significantly higher among the late subtypes (145, (71%)) vs. early (51 (45%), *P* = < 0.001) or NA subtypes (48, (55%), *P* = 0.01), as illustrated in S4B Fig. In addition to mIgVH, ZAP-70 status was negative in 65% of ME patients (Table 3), which fits with their (general) more favorable phenotype [34][35]. However, nine patients assigned to the ME subtype carried del17p (11%), which is linked to a poorer outcome. Comparing the cytogenetic and IgVH status of these ME patients, we found that six carried uIgVH. Despite, a high proportion of patients aged < = 65 in the BII subgroup (90.1%), no significant differences were observed between subtypes (Table 3). In line with the literature, males constituted the larger proportion of diagnosed patients (61%; Table 3), although the IM and PC patient group included more female patients (58% and 59%; Table 3).

**Table 3:** Associations between BAGS subtypes and patient characteristics, based on available clinical data.

	Pre-BI	Pre-BII	Immature	Naïve	Memory	Plasma Cell	Total
**Age**							
< = 65 years	21 (13.8)	10 (6.6)	8 (5.3)	44 (28.9)	64 (42.1)	5 (3.3)	152
>65 years	19 (18.4)	1 (1.0)	4 (3.9)	23 (22.3)	50 (48.5)	6 (5.8)	103
**Gender**							
Female	21 (12.4)	5 (3.0)	18 (10.7)	32 (18.9)	83 (49.1)	10 (5.9)	169
Male	45 (17.2)	10 (3.8)	13 (5.0)	68 (26.1)	118 (45.2)	7 (2.9)	261
**Binet stage**							
A	4 (8.7)	1 (2.2)	4 (8.7)	14 (30.4)	18 (39.1)	5 (10.9)	46
B-C	7 (25.0)	1 (3.6)	0 (0.0)	12 (42.9)	7 (25.0)	1 (3.6)	28
**Cytogenetic status**							
No marker	7 (15.6)	2 (4.4)	2 (4.4)	8 (17.8)	22 (48.9)	4 (8.9)	45
Del13q	16 (16.2)	3 (3.0)	4 (4.0)	26 (26.3)	44 (44.4)	6 (6.1)	99
Tri12	7 (25.0)	0 (0.0)	3 (10.7)	3 (10.7)	14 (50)	1 (3.6)	28
Del11q	4 (21.1)	0 (0.0)	2 (10.5)	9 (47.4)	4 (21.1)	0 (0.0)	19
Del17p	0 (0.0)	0 (0.0)	1 (8.3)	1 (8.3)	9 (75.0)	1 (8.3)	12
**ZAP-70 status**							
Positive	24 (20.9)	3 (2.6)	14 (12.2)	22 (19.1)	48 (41.7)	4 (3.5)	115
Negative	20 (12.1)	5 (3.0)	15 (9.1)	32 (19.4)	89 (54.0)	4 (2.4)	165
**IgVH status**							
uIgVH	35 (21.7)	8 (5.0)	20 (12.4)	39 (24.2)	53 (32.9)	6 (3.7)	161
mIgVH	31 (12.7)	5 (2.0)	15 (6.1)	48 (19.7)	135 (55.3)	10 (4.1)	244

Abbreviations: Del13q, deletion of 13q; Tri12, Trisomy; Del17p, deletion of 17p; Del11q, deletion of 11q; ZAP-70, zeta-chain-associated protein kinase 70; IgVH, immunoglobulin variable region heavy chain; mIgVH, mutated IgVH; uIgVH, unmutated IgVH.

### Predictive drug resistance in CLL subtypes

To further investigate the prognostic impact of BAGS, we pursued an indirect approach using predicted resistance to R and C in the CLL samples. As a reference for malignant samples, we also assessed the inherent resistance levels predicted for normal B-cell subsets.

**Fig 3:**
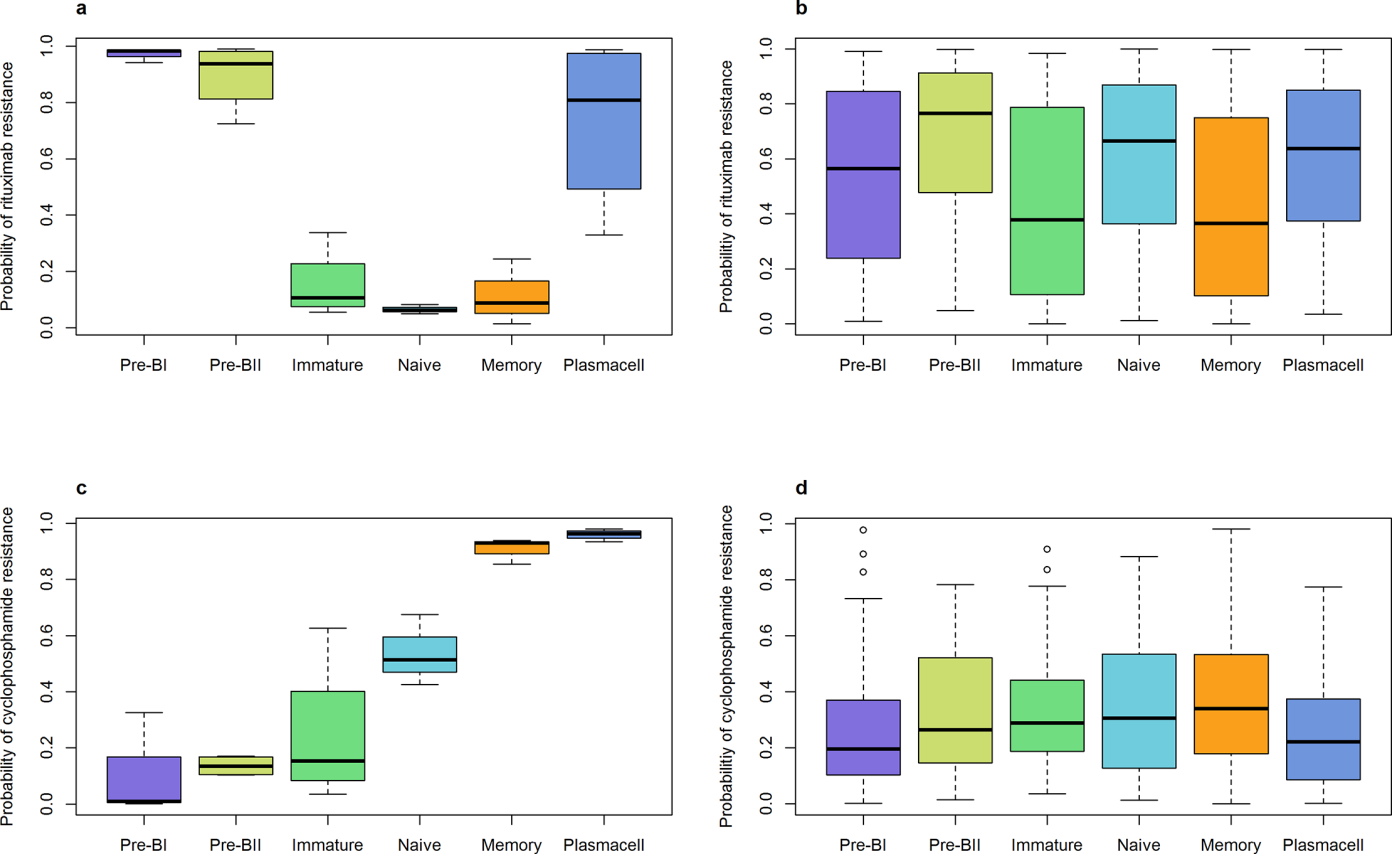
Drug resistance to rituximab or cyclophosphamide. Box plots represent the estimated probability of resistance to (**a**) Rituximab in normal B-cell subsets, (**b**) Rituximab in all CLL samples, (**c**) Cyclophosphamide in normal B-cell subsets and (**d**) Cyclophosphamide in all CLL samples. The global *P*-value for equal mean resistance probability was < 0.001 in all four cases. Color codes as in Fig 1.

We observed a high probability of resistance to R in the normal BI, BII, and PC subsets, whereas a low resistance probability was seen in the IM, NA, and ME subsets (*P* < .001) (Fig 3A). Variability in R resistance was observed across the CLL subtypes (*P* < .001) (Fig 3B), with the IM and ME subtypes demonstrating the least probability of resistance. The BII subtype had the highest probability of resistance, suggesting that this subtype not only exhibits a shorter TTT compared to the other subtypes, but possibly also an inferior prognosis following therapy. The probability of resistance to C was found to increase with differentiation stage in the normal B-cell subsets (*P* < .001), such that ME and PC showed the highest probabilities of resistance (Fig 3C). However, for all CLL subtypes, a comparable sensitivity pattern to C, with no substantial differences in predicted resistance level, was observed (Fig 3D).

## Discussion

In the present study, FACS, GEP, and statistical modeling were combined to create a classifier that could categorize CLL samples according to BAGS into pre-BI, pre-BII, immature, naïve, memory, or plasma cell subtypes. We hypothesized that this subtyping would have prognostic and thus biological implications in CLL. Subsequently we documented that CLL patients with an early BAGS phenotype manifested significantly shorter TTT's vs. CLL patients with late BAGS phenotypes, with the pre-BII subtype appearing to be the least favorable.

Our concept is based on a phenotypic cell-of-origin approach, which has previously been applied to diffuse large B-cell lymphoma based on GEP of normal tonsil B-cell subsets. It remains an open question as to whether the phenotype of malignant cells in CLL authentically reflects features of maturation steps, including direct precursors. Tumorigenesis is a multi-step process and the first transforming events in CLL may arise in early differentiation stages, possibly even in hematopoietic stem cells [36]. In the event of an initial genomic hit targeting a less differentiated B-cell, the principal transcriptional program of each end-stage tumor still likely reflects some aspects of normal B-cell phenotypes. Further, the high frequency of the NA and ME subtypes found here agrees with previous studies. Klein et al. found that CLL generally resembles memory B-cells more closely than either naïve B-cells, CD5^+^ B-cells, germinal center centroblasts, or centrocytes [37]. Seifert et al. reported a higher similarity of CLL to naïve B-cells and determined that the direct precursor of the CLL clone is an antigen-exposed CD5^+^ B-cell, irrespective of IgVH mutation status, that results in the production of mono- or oligoclonal B-cells [38].

Interestingly, we found that mIgVH was more frequent among late subtypes than early. Somatic hypermutation occurs in the germinal center or similar structures, and it could be argued that any CLL bearing mutated IgVH must have undergone antigenic selection and therefore stems from a post-germinal subtype [32].

It is notable that the BAGS classifier for ME subtypes exhibited low CD38 (S5 Table) as this marker is found down-regulated in normal mature B-cells.

Low CD38 correlates highly with a largely negative ZAP-70 status and mIgVH in CLL ME patients, who also manifest a longer TTT compared to earlier subtypes.

Despite indications of a hierarchical pattern of association between BAGS and IgVH, samples with either mutation status (mutated/unmutated) were observed for each subtype. To that end, the presence of early pregerminal subtypes may indicate a reversible phenotypic plasticity [39–40] in CLL that we have yet to further explore.

Further supportive data are now required if we are to achieve a diagnostic phenotyping capability that can support individualized therapy. These studies will necessarily involve a range of technical, statistical, and clinical considerations, as detailed below.

### Technical and statistical considerations

We used sternal BM tissue for the isolation and analysis of B-cell subset compartments. Normal B-cell subsets from BM were successfully sorted and prepared for microarray analyses as previously described.^13^ Data quality was validated by hierarchical clustering with PCA allowing us to generate six BAGS, one per major B-cell compartment in the BM.

Penalized multinomial logistic regression was used to assign each CLL sample to one of six BAGS subtypes. BAGS assignments for 85% of CLL samples were achieved, with a reasonable probability cut-off across the cohorts (ranging from 0.38 to 0.50). Our methodology included cohort-based normalization, median centering, and scaling of gene-wise variance prior to BAGS classification. Therefore, in its current form, "cohort-based" BAGS profiling cannot be applied to individual patients as would be required in the clinical setting. However, in a recent study, this caveat was overcome by inferring a one-by-one microarray normalization scheme, which enabled the classification of individual samples generated on the HG-U133plus2 gene array [41]. A similar approach is under consideration for the current study.

In this study, sorted samples hybridized to Exon arrays were used to generate BAGS, while clinical samples were hybridized to the HG-U133plus2 array. It has been shown that these two platforms have a similar ability to distinguish between differentially and non-differentially expressed genes [42]. In addition, platform compatibility was validated by the 100% accuracy with which normal healthy (sorted) samples were categorized following hybridization to HG-U133plus2 arrays, illustrating methodological robustness across platforms of the BAGS classifier signature genes. We therefore found it acceptable to apply the exon array-based classifier to material hybridized to other array platforms.

### Clinical considerations

This study included untreated patient samples from seven different centers with different research goals. Clinical data were available for a limited number of participants (S3 Table), although sufficient material was available to study the association of BAGS to TTT and IgVH status. Our preference, to investigate the prognostic impact of BAGS on overall survival, was precluded by insufficient patient data together with favorable CLL prognoses and therefore few events. This endpoint warrants further future study in more suitable cohorts.

The investigated CLL cells in this study stem from peripheral blood (PB) draws, as CLL diagnosis in the clinical setting is based on routine samples from PB, with avoidance of invasive tests such as BM and lymph node biopsies. It has previously been shown that CLL gene expression differs between PB, BM, and lymph nodes [43]. Proliferation has been shown to predominantly occur in secondary lymphoid organs where the microenvironment facilitates survival and disease progression [44], while circulating tumor cells in PB display a more resting phenotype [45]. It was of primary interest to utilize B-cell subsets from BM as it enabled investigation of early B-cell subset signatures. However, it could be of further interest to investigate whether BAGS classifications differ according to the sampling site, a possibility that has not been pursued here.

The majority of included samples relied on purified CLL cells, while the Munich and Sapienza cohorts were based on unpurified peripheral blood. However, the distribution of BAGS subtypes was similar to the other cohorts (S3 Fig) and a sensitivity analysis of TTT, where the Munich cohort was excluded, showed only minor changes in the cumulative incidence trajectories (S5 Fig). Fewer statistically significant results were obtained in the sensitivity analysis, which likely was due to reduced power caused by the lower sample number.

As a surrogate for post-treatment prognosis, REGS classifications were applied to the CLL samples with respect to R and C. Standard first-line treatment of CLL comprises a combined immune- and chemotherapeutic approach for patients without 17p- and/or inactivating TP53 mutations, often the FCR (fludarabine, cyclophosphamide, and rituximab) regimen [46–49]. The diverse resistance probabilities seen for R, of which the pre-BII subtypes were predicted to be most resistant, suggests that BAGS classification data may be of some prognostic value post-treatment. Fischer et al. found that among patients treated with FCR, long-term remission was most likely in patients with mIgVH [50]. This may also be achieved for patients with different BAGS subtypes. For example, the ME subtypes (predominantly mIgVH) were also predicted to be the least resistant to R. These findings should now be validated in studies of post-treatment progression-free follow-up.

Here we have retrospectively studied CLL samples for phenotypic differences assigned by normal BM B-cell subset gene signatures. The main finding was that BAGS classification demonstrated a prognostic association with TTT. Critically, the classification included pre-germinal subtypes, indicating a reversible phenotypic plasticity in leukemic B cells. Future prospective studies will attempt to prove the concept by clinical end-points following treatment, including prognosis and the prediction of therapeutic outcome.

## Supporting information

S1 Appendix Supporting text. (PDF)

S1 Table Highly selected monoclonal antibody panel with which to immunophenotype bone marrow-derived B-cell subsets. (PDF)

S2 Table List of included CLL patients in the study. (PDF)

S3 Table Characteristics of the CLL Cohorts included in the study. (PDF)

S4 Table Agreement between array platforms tested using normal B-cell subsets. (PDF)

S5 Table BAGS classifier genes. (PDF)

S6 Table BAGS assignment, clinical variables, and outcome. (PDF)

S1 Fig Time to first treatment in the Munich, UCSD, and IIDFCI cohorts. (PDF)

S2 Fig Assigned CLL samples and probabilities of association to BAGS subtypes. (PDF)

S3 Fig BAGS subtype distribution across the eight CLL cohorts. (PDF)

S4 Fig Association between IgVH mutation status and BAGS subtype. (PDF)

S5 Fig Analyses of the prognostic impact of subtyping according to BAGS on TTT in watch-and-wait CLL. (PDF)
