# Supplementary material for: Subtype assignment of CLL based on B-cell subset associated gene signatures from normal bone marrow – A proof of concept study
Source: PLoS One. 2018 Mar 7;13(3):e0193249. doi: 10.1371/journal.pone.0193249 (PMC5841735; doi:10.1371/journal.pone.0193249)
Supplement: S1 Appendix — (PDF) [file pone.0193249.s001.pdf]

Supporting Text

Hypothesis and aims:

Following a prospective systematic approach, we propose a classification system that includes subset specific B-cell associated gene signatures (BAGS) extracted from the normal B-cell hierarchy.

The present hypothesis is that BAGS based on six naturally occurring B-cell subsets in normal bone marrow can be used to assign CLL subtypes of prognostic and pathogenetic value. The aims are to:

1) Qualify the gene expression profiles in the six sorted B-cell subsets;
2) Generate a useful and validated BAGS classifier;
3) Estimate the frequencies of CLL subtypes in available patient cohorts;
4) Document the prognostic impact of BAGS subtyping in CLL.

Ethical aspects and tissue preparation

All normal samples were processed in accordance with the scientific protocol accepted by the local ethical committee (N-20080062MCH). Healthy sternal bone marrow was surgically harvested from seven patients who underwent cardiac surgery. The tissue was sectioned and homogenized in phosphate buffered saline (PBS) using a syringe. Tissue suspensions were washed once in PBS, with mononuclear cells (MNC) subsequently enriched by gradient centrifugation using Ficoll-Paque Plus (GE Health Care, Uppsala, Sweden) in accordance with the manufacturer's instructions. Bone marrow was homogenized in 2 ml PBS using a syringe. Red blood cells were lysed with 20 ml of Easylyse (DAKO, Glostrup, Denmark) and incubated for 45 min at room temperature. Samples were washed once in PBS and then filtered (40μM filter) to remove debris and aggregates.

Multiparametric Flow Cytometry (MFC)

Briefly, MNC from sternal bone marrow were stained with an 7 colour panel of monoclonal antibodies (mAb) (as listed in S1 Table and described previously[12] and incubated in stain buffer (BD Biosciences, San Diego, CA) for 30 min at room temperature (in the dark). Stained cells were washed and resuspended in stain buffer and sorted immediately using a BD FACSAria2 cell sorter. FACSDiva software (BD Biosciences, San Jose, CA) was used for MFC-based identification and gating. Compensation was automatically calculated using control single antibody stained samples. Cells were filtered (35µm filter; cell strainer, BD Biosciences) immediately before data acquisition. Purity of the isolated B-cell subsets (>90%) was confirmed by sorting approximately 1000 cells into PBS followed by reacquisition of the sorted B-cell subsets. Cells were sorted into 450 µl lysis/binding buffer (Miltenyi Biotech, Bergisch-Gladbach, Germany) then stored at -20 °C.

CLL data sets

The NCBI Gene Expression Omnibus (repository) was queried for all relevant gene expression data sets generated on the Affymetrix Human Genome U133 plus 2.0 arrays. The search (performed June 25th 2015), conducted for "Homo Sapiens" samples, included the search terms: "(("CLL"[All Fields] OR "Chronic Lymphatic Leukemia"[All Fields] OR "Chronic Lymphocytic Leukemia"[All Fields] OR "Chronic B Lymphocytic Leukemia"[All Fields] OR "Chronic B-Lymphocytic Leukemia"[All Fields] OR "B-Cell Chronic Lymphocytic Leukemia"[All Fields])) AND (((prognosis) OR outcome) OR surviv*)". We retrieved 271 hits. Next, we queried the repository for any datasets missed in the first search using the search terms: "CLL"[All Fields] OR "Chronic Lymphatic Leukemia"[All Fields] OR "Chronic Lymphocytic Leukemia"[All Fields] OR "Chronic B Lymphocytic Leukemia"[All Fields] OR "Chronic B-Lymphocytic Leukemia"[All Fields] OR "B-Cell Chronic Lymphocytic Leukemia"[All Fields]. One

hundred and ninety-four hits were retrieved. Data sets with 50 samples or more were included. The search was repeated on April 17th 2016 to capture any newly deposited data sets.

Initially, eleven data sets were included; those listed as normal were then excluded. Furthermore, treatment information for patients in respective cohorts were searched for using cohort descriptions and sample names in the Gene Expression Omnibus, as well as in the listed articles. The MD5 algorithm was used to detect patient overlap between cohorts. This resulted in the eight final cohorts that we included. Patient numbers and GSE accession numbers for each cohort are shown in S2 Table.
