# Supplementary material for: Subtype assignment of CLL based on B-cell subset associated gene signatures from normal bone marrow – A proof of concept study
Source: PLoS One. 2018 Mar 7;13(3):e0193249. doi: 10.1371/journal.pone.0193249 (PMC5841735; doi:10.1371/journal.pone.0193249)
Supplement: S1 Table — (PDF) [file pone.0193249.s002.pdf]

S1 Table. Highly selected monoclonal antibody panel with which to immunophenotype bone marrow-derived B-cell subsets

	CD10	CD19	CD20	CD27	CD34	CD38	CD45
**Pre-BI**	++	+	-	-	+	++	-d
**Pre-BII**	+	+	het	-	-	+	+
**Immature**	+	+	+	-	-	+	+
**Naïve**	-	+	+	-	-	-	+
**Memory**	-	+	+	+	-	-	+
**Plasma cell**	-	het	-	++	-	+++	het

NOTE: Panel of differentiation marker combinations used to categorize B-cell subsets. ++ = strongly positive, + = positive, - = negative, -d = dimly positive, het = heterogenous.
