# Supplementary material for: Subtype assignment of CLL based on B-cell subset associated gene signatures from normal bone marrow – A proof of concept study
Source: PLoS One. 2018 Mar 7;13(3):e0193249. doi: 10.1371/journal.pone.0193249 (PMC5841735; doi:10.1371/journal.pone.0193249)
Supplement: S2 Table — (PDF) [file pone.0193249.s003.pdf]

S2 Table. List of included CLL patients in the study.

GEO_ID	Study	GSE_ID	GEO_ID	Study	GSE_ID
GSM877692	SAPIENZA	GSE69034	GSM331287	ROCHE	GSE39671
GSM877693	SAPIENZA	GSE69034	GSM331288	ROCHE	GSE39671
GSM877694	SAPIENZA	GSE69034	GSM331289	ROCHE	GSE39671
GSM877695	SAPIENZA	GSE69034	GSM331290	ROCHE	GSE39671
GSM877696	SAPIENZA	GSE69034	GSM331291	ROCHE	GSE39671
GSM877697	SAPIENZA	GSE69034	GSM331292	ROCHE	GSE39671
GSM877698	SAPIENZA	GSE69034	GSM331293	ROCHE	GSE39671
GSM877699	SAPIENZA	GSE69034	GSM331294	ROCHE	GSE39671
GSM877700	SAPIENZA	GSE69034	GSM331295	ROCHE	GSE39671
GSM877701	SAPIENZA	GSE69034	GSM331296	ROCHE	GSE39671
GSM877702	SAPIENZA	GSE69034	GSM331298	ROCHE	GSE39671
GSM877703	SAPIENZA	GSE69034	GSM331300	ROCHE	GSE39671
GSM877704	SAPIENZA	GSE69034	GSM331301	ROCHE	GSE39671
GSM877705	SAPIENZA	GSE69034	GSM331302	ROCHE	GSE39671
GSM877706	SAPIENZA	GSE69034	GSM331304	ROCHE	GSE39671
GSM877707	SAPIENZA	GSE69034	GSM331305	ROCHE	GSE39671
GSM877708	SAPIENZA	GSE69034	GSM331306	ROCHE	GSE39671
GSM877709	SAPIENZA	GSE69034	GSM331307	ROCHE	GSE39671
GSM877710	SAPIENZA	GSE69034	GSM331309	ROCHE	GSE39671
GSM877711	SAPIENZA	GSE69034	GSM331312	ROCHE	GSE39671
GSM877712	SAPIENZA	GSE69034	GSM331313	ROCHE	GSE39671
GSM877713	SAPIENZA	GSE69034	GSM331314	ROCHE	GSE39671
GSM877714	SAPIENZA	GSE69034	GSM331315	ROCHE	GSE39671
GSM877715	SAPIENZA	GSE69034	GSM331316	ROCHE	GSE39671
GSM877716	SAPIENZA	GSE69034	GSM331317	ROCHE	GSE39671
GSM877717	SAPIENZA	GSE69034	GSM331320	ROCHE	GSE39671
GSM877718	SAPIENZA	GSE69034	GSM331321	ROCHE	GSE39671
GSM877719	SAPIENZA	GSE69034	GSM331322	ROCHE	GSE39671
GSM877720	SAPIENZA	GSE69034	GSM331327	ROCHE	GSE39671
GSM877721	SAPIENZA	GSE69034	GSM331328	ROCHE	GSE39671
GSM877722	SAPIENZA	GSE69034	GSM331329	ROCHE	GSE39671
GSM877723	SAPIENZA	GSE69034	GSM331330	ROCHE	GSE39671
GSM877724	SAPIENZA	GSE69034	GSM331331	ROCHE	GSE39671

GSM877725	SAPIENZA	GSE69034	GSM331332	ROCHE	GSE39671
GSM877726	SAPIENZA	GSE69034	GSM331333	ROCHE	GSE39671
GSM877727	SAPIENZA	GSE69034	GSM331334	ROCHE	GSE39671
GSM877728	SAPIENZA	GSE69034	GSM331336	ROCHE	GSE39671
GSM877729	SAPIENZA	GSE69034	GSM331339	ROCHE	GSE39671
GSM877730	SAPIENZA	GSE69034	GSM331340	ROCHE	GSE39671
GSM877731	SAPIENZA	GSE69034	GSM331342	ROCHE	GSE39671
GSM877732	SAPIENZA	GSE69034	GSM331343	ROCHE	GSE39671
GSM877733	SAPIENZA	GSE69034	GSM331344	ROCHE	GSE39671
GSM877734	SAPIENZA	GSE69034	GSM331347	ROCHE	GSE39671
GSM877735	SAPIENZA	GSE69034	GSM331348	ROCHE	GSE39671
GSM877736	SAPIENZA	GSE69034	GSM331350	ROCHE	GSE39671
GSM877737	SAPIENZA	GSE69034	GSM331351	ROCHE	GSE39671
GSM877738	SAPIENZA	GSE69034	GSM331352	ROCHE	GSE39671
GSM877739	SAPIENZA	GSE69034	GSM331354	ROCHE	GSE39671
GSM877740	SAPIENZA	GSE69034	GSM331358	ROCHE	GSE39671
GSM877741	SAPIENZA	GSE69034	GSM331359	ROCHE	GSE39671
GSM877742	SAPIENZA	GSE69034	GSM331362	ROCHE	GSE39671
GSM877743	SAPIENZA	GSE69034	GSM331363	ROCHE	GSE39671
GSM877744	SAPIENZA	GSE69034	GSM331364	ROCHE	GSE39671
GSM877745	SAPIENZA	GSE69034	GSM331365	ROCHE	GSE39671
GSM877746	SAPIENZA	GSE69034	GSM331366	ROCHE	GSE39671
GSM877747	SAPIENZA	GSE69034	GSM331368	ROCHE	GSE39671
GSM877748	SAPIENZA	GSE69034	GSM331369	ROCHE	GSE39671
GSM877749	SAPIENZA	GSE69034	GSM331370	ROCHE	GSE39671
GSM877750	SAPIENZA	GSE69034	GSM331371	ROCHE	GSE39671
GSM877751	SAPIENZA	GSE69034	GSM331372	ROCHE	GSE39671
GSM877752	SAPIENZA	GSE69034	GSM331373	ROCHE	GSE39671
GSM877753	SAPIENZA	GSE69034	GSM331374	ROCHE	GSE39671
GSM256312	DUKE	GSE35935	GSM331376	ROCHE	GSE39671
GSM256313	DUKE	GSE35935	GSM709859	PADOVA	GSE28654
GSM256314	DUKE	GSE35935	GSM709860	PADOVA	GSE28654
GSM256284	DUKE	GSE35935	GSM709861	PADOVA	GSE28654
GSM256285	DUKE	GSE35935	GSM709862	PADOVA	GSE28654
GSM256315	DUKE	GSE35935	GSM709863	PADOVA	GSE28654

GSM256265	DUKE	GSE35935	GSM709864	PADOVA	GSE28654
GSM256286	DUKE	GSE35935	GSM709865	PADOVA	GSE28654
GSM256287	DUKE	GSE35935	GSM709866	PADOVA	GSE28654
GSM256316	DUKE	GSE35935	GSM709867	PADOVA	GSE28654
GSM256317	DUKE	GSE35935	GSM709868	PADOVA	GSE28654
GSM256288	DUKE	GSE35935	GSM709869	PADOVA	GSE28654
GSM256318	DUKE	GSE35935	GSM709870	PADOVA	GSE28654
GSM256319	DUKE	GSE35935	GSM709871	PADOVA	GSE28654
GSM256289	DUKE	GSE35935	GSM709872	PADOVA	GSE28654
GSM256290	DUKE	GSE35935	GSM709873	PADOVA	GSE28654
GSM256291	DUKE	GSE35935	GSM709874	PADOVA	GSE28654
GSM256320	DUKE	GSE35935	GSM709875	PADOVA	GSE28654
GSM256292	DUKE	GSE35935	GSM709876	PADOVA	GSE28654
GSM256321	DUKE	GSE35935	GSM709877	PADOVA	GSE28654
GSM256293	DUKE	GSE35935	GSM709878	PADOVA	GSE28654
GSM256294	DUKE	GSE35935	GSM709879	PADOVA	GSE28654
GSM256295	DUKE	GSE35935	GSM709880	PADOVA	GSE28654
GSM256296	DUKE	GSE35935	GSM709881	PADOVA	GSE28654
GSM256297	DUKE	GSE35935	GSM709882	PADOVA	GSE28654
GSM256322	DUKE	GSE35935	GSM709883	PADOVA	GSE28654
GSM256323	DUKE	GSE35935	GSM709884	PADOVA	GSE28654
GSM256324	DUKE	GSE35935	GSM709885	PADOVA	GSE28654
GSM256266	DUKE	GSE35935	GSM709886	PADOVA	GSE28654
GSM256325	DUKE	GSE35935	GSM709887	PADOVA	GSE28654
GSM256326	DUKE	GSE35935	GSM709888	PADOVA	GSE28654
GSM256327	DUKE	GSE35935	GSM709889	PADOVA	GSE28654
GSM256328	DUKE	GSE35935	GSM709890	PADOVA	GSE28654
GSM256329	DUKE	GSE35935	GSM709891	PADOVA	GSE28654
GSM256330	DUKE	GSE35935	GSM709892	PADOVA	GSE28654
GSM256331	DUKE	GSE35935	GSM709893	PADOVA	GSE28654
GSM256298	DUKE	GSE35935	GSM709894	PADOVA	GSE28654
GSM256299	DUKE	GSE35935	GSM709895	PADOVA	GSE28654
GSM256267	DUKE	GSE35935	GSM709896	PADOVA	GSE28654
GSM256268	DUKE	GSE35935	GSM709897	PADOVA	GSE28654
GSM256269	DUKE	GSE35935	GSM709898	PADOVA	GSE28654

GSM256270	DUKE	GSE35935	GSM709899	PADOVA	GSE28654
GSM256271	DUKE	GSE35935	GSM709900	PADOVA	GSE28654
GSM256272	DUKE	GSE35935	GSM709901	PADOVA	GSE28654
GSM256300	DUKE	GSE35935	GSM709902	PADOVA	GSE28654
GSM256301	DUKE	GSE35935	GSM709903	PADOVA	GSE28654
GSM256273	DUKE	GSE35935	GSM709904	PADOVA	GSE28654
GSM256302	DUKE	GSE35935	GSM709905	PADOVA	GSE28654
GSM256303	DUKE	GSE35935	GSM709906	PADOVA	GSE28654
GSM256264	DUKE	GSE35935	GSM709907	PADOVA	GSE28654
GSM256304	DUKE	GSE35935	GSM709908	PADOVA	GSE28654
GSM256305	DUKE	GSE35935	GSM709909	PADOVA	GSE28654
GSM256274	DUKE	GSE35935	GSM709910	PADOVA	GSE28654
GSM256275	DUKE	GSE35935	GSM709911	PADOVA	GSE28654
GSM256276	DUKE	GSE35935	GSM709912	PADOVA	GSE28654
GSM256306	DUKE	GSE35935	GSM709913	PADOVA	GSE28654
GSM256277	DUKE	GSE35935	GSM709914	PADOVA	GSE28654
GSM256307	DUKE	GSE35935	GSM709915	PADOVA	GSE28654
GSM256278	DUKE	GSE35935	GSM709916	PADOVA	GSE28654
GSM256308	DUKE	GSE35935	GSM709917	PADOVA	GSE28654
GSM256279	DUKE	GSE35935	GSM709918	PADOVA	GSE28654
GSM256280	DUKE	GSE35935	GSM709919	PADOVA	GSE28654
GSM256281	DUKE	GSE35935	GSM709920	PADOVA	GSE28654
GSM256309	DUKE	GSE35935	GSM709921	PADOVA	GSE28654
GSM256310	DUKE	GSE35935	GSM709922	PADOVA	GSE28654
GSM256282	DUKE	GSE35935	GSM709923	PADOVA	GSE28654
GSM256283	DUKE	GSE35935	GSM709924	PADOVA	GSE28654
GSM256311	DUKE	GSE35935	GSM709925	PADOVA	GSE28654
GSM562854	MUNICH	GSE13204	GSM709926	PADOVA	GSE28654
GSM562948	MUNICH	GSE13204	GSM709927	PADOVA	GSE28654
GSM562949	MUNICH	GSE13204	GSM709928	PADOVA	GSE28654
GSM562950	MUNICH	GSE13204	GSM709929	PADOVA	GSE28654
GSM562951	MUNICH	GSE13204	GSM709930	PADOVA	GSE28654
GSM562856	MUNICH	GSE13204	GSM709931	PADOVA	GSE28654
GSM562952	MUNICH	GSE13204	GSM709932	PADOVA	GSE28654
GSM562953	MUNICH	GSE13204	GSM709933	PADOVA	GSE28654

GSM562954	MUNICH	GSE13204	GSM709934	PADOVA	GSE28654
GSM562955	MUNICH	GSE13204	GSM709935	PADOVA	GSE28654
GSM562858	MUNICH	GSE13204	GSM709936	PADOVA	GSE28654
GSM562957	MUNICH	GSE13204	GSM709937	PADOVA	GSE28654
GSM562860	MUNICH	GSE13204	GSM709938	PADOVA	GSE28654
GSM562958	MUNICH	GSE13204	GSM709939	PADOVA	GSE28654
GSM562959	MUNICH	GSE13204	GSM709940	PADOVA	GSE28654
GSM562960	MUNICH	GSE13204	GSM709941	PADOVA	GSE28654
GSM562861	MUNICH	GSE13204	GSM709942	PADOVA	GSE28654
GSM562862	MUNICH	GSE13204	GSM709943	PADOVA	GSE28654
GSM562961	MUNICH	GSE13204	GSM709944	PADOVA	GSE28654
GSM562962	MUNICH	GSE13204	GSM709945	PADOVA	GSE28654
GSM562963	MUNICH	GSE13204	GSM709946	PADOVA	GSE28654
GSM562964	MUNICH	GSE13204	GSM709947	PADOVA	GSE28654
GSM562863	MUNICH	GSE13204	GSM709948	PADOVA	GSE28654
GSM562965	MUNICH	GSE13204	GSM709949	PADOVA	GSE28654
GSM562864	MUNICH	GSE13204	GSM709950	PADOVA	GSE28654
GSM562968	MUNICH	GSE13204	GSM709951	PADOVA	GSE28654
GSM562969	MUNICH	GSE13204	GSM709952	PADOVA	GSE28654
GSM562866	MUNICH	GSE13204	GSM709953	PADOVA	GSE28654
GSM562867	MUNICH	GSE13204	GSM709954	PADOVA	GSE28654
GSM562868	MUNICH	GSE13204	GSM709955	PADOVA	GSE28654
GSM562869	MUNICH	GSE13204	GSM709956	PADOVA	GSE28654
GSM562971	MUNICH	GSE13204	GSM709957	PADOVA	GSE28654
GSM562870	MUNICH	GSE13204	GSM709958	PADOVA	GSE28654
GSM562871	MUNICH	GSE13204	GSM709959	PADOVA	GSE28654
GSM562972	MUNICH	GSE13204	GSM709960	PADOVA	GSE28654
GSM562973	MUNICH	GSE13204	GSM709961	PADOVA	GSE28654
GSM562874	MUNICH	GSE13204	GSM709962	PADOVA	GSE28654
GSM562974	MUNICH	GSE13204	GSM709963	PADOVA	GSE28654
GSM562975	MUNICH	GSE13204	GSM709964	PADOVA	GSE28654
GSM562875	MUNICH	GSE13204	GSM709965	PADOVA	GSE28654
GSM562876	MUNICH	GSE13204	GSM709966	PADOVA	GSE28654
GSM562976	MUNICH	GSE13204	GSM709967	PADOVA	GSE28654
GSM562977	MUNICH	GSE13204	GSM709968	PADOVA	GSE28654

GSM562978	MUNICH	GSE13204	GSM709969	PADOVA	GSE28654
GSM562877	MUNICH	GSE13204	GSM709970	PADOVA	GSE28654
GSM562979	MUNICH	GSE13204	GSM977137	UCSD	GSE50006
GSM562980	MUNICH	GSE13204	GSM977138	UCSD	GSE50006
GSM562982	MUNICH	GSE13204	GSM977139	UCSD	GSE50006
GSM562879	MUNICH	GSE13204	GSM977140	UCSD	GSE50006
GSM562983	MUNICH	GSE13204	GSM977141	UCSD	GSE50006
GSM562880	MUNICH	GSE13204	GSM977142	UCSD	GSE50006
GSM562984	MUNICH	GSE13204	GSM977143	UCSD	GSE50006
GSM562985	MUNICH	GSE13204	GSM977144	UCSD	GSE50006
GSM562881	MUNICH	GSE13204	GSM977145	UCSD	GSE50006
GSM562986	MUNICH	GSE13204	GSM977146	UCSD	GSE50006
GSM562987	MUNICH	GSE13204	GSM977147	UCSD	GSE50006
GSM562988	MUNICH	GSE13204	GSM977148	UCSD	GSE50006
GSM562989	MUNICH	GSE13204	GSM977149	UCSD	GSE50006
GSM562990	MUNICH	GSE13204	GSM977150	UCSD	GSE50006
GSM562991	MUNICH	GSE13204	GSM977151	UCSD	GSE50006
GSM562992	MUNICH	GSE13204	GSM977152	UCSD	GSE50006
GSM562882	MUNICH	GSE13204	GSM977153	UCSD	GSE50006
GSM562993	MUNICH	GSE13204	GSM977154	UCSD	GSE50006
GSM562994	MUNICH	GSE13204	GSM977155	UCSD	GSE50006
GSM562995	MUNICH	GSE13204	GSM977156	UCSD	GSE50006
GSM562996	MUNICH	GSE13204	GSM977157	UCSD	GSE50006
GSM562883	MUNICH	GSE13204	GSM977158	UCSD	GSE50006
GSM562884	MUNICH	GSE13204	GSM977159	UCSD	GSE50006
GSM562998	MUNICH	GSE13204	GSM977160	UCSD	GSE50006
GSM562999	MUNICH	GSE13204	GSM977161	UCSD	GSE50006
GSM563000	MUNICH	GSE13204	GSM977162	UCSD	GSE50006
GSM563001	MUNICH	GSE13204	GSM977163	UCSD	GSE50006
GSM563004	MUNICH	GSE13204	GSM977164	UCSD	GSE50006
GSM563005	MUNICH	GSE13204	GSM977165	UCSD	GSE50006
GSM563006	MUNICH	GSE13204	GSM977166	UCSD	GSE50006
GSM563007	MUNICH	GSE13204	GSM977167	UCSD	GSE50006
GSM563008	MUNICH	GSE13204	GSM977168	UCSD	GSE50006
GSM563010	MUNICH	GSE13204	GSM977169	UCSD	GSE50006

GSM563011	MUNICH	GSE13204	GSM977170	UCSD	GSE50006
GSM562885	MUNICH	GSE13204	GSM977171	UCSD	GSE50006
GSM563012	MUNICH	GSE13204	GSM977172	UCSD	GSE50006
GSM563013	MUNICH	GSE13204	GSM977173	UCSD	GSE50006
GSM563014	MUNICH	GSE13204	GSM977174	UCSD	GSE50006
GSM563015	MUNICH	GSE13204	GSM977175	UCSD	GSE50006
GSM563016	MUNICH	GSE13204	GSM977176	UCSD	GSE50006
GSM563017	MUNICH	GSE13204	GSM977177	UCSD	GSE50006
GSM562886	MUNICH	GSE13204	GSM977178	UCSD	GSE50006
GSM563018	MUNICH	GSE13204	GSM977179	UCSD	GSE50006
GSM563020	MUNICH	GSE13204	GSM977180	UCSD	GSE50006
GSM563021	MUNICH	GSE13204	GSM977181	UCSD	GSE50006
GSM563022	MUNICH	GSE13204	GSM977182	UCSD	GSE50006
GSM563023	MUNICH	GSE13204	GSM977183	UCSD	GSE50006
GSM563024	MUNICH	GSE13204	GSM977184	UCSD	GSE50006
GSM562887	MUNICH	GSE13204	GSM977185	UCSD	GSE50006
GSM563025	MUNICH	GSE13204	GSM977186	UCSD	GSE50006
GSM563026	MUNICH	GSE13204	GSM977187	UCSD	GSE50006
GSM563027	MUNICH	GSE13204	GSM977188	UCSD	GSE50006
GSM563028	MUNICH	GSE13204	GSM977189	UCSD	GSE50006
GSM563029	MUNICH	GSE13204	GSM977190	UCSD	GSE50006
GSM563030	MUNICH	GSE13204	GSM977191	UCSD	GSE50006
GSM563031	MUNICH	GSE13204	GSM977192	UCSD	GSE50006
GSM563032	MUNICH	GSE13204	GSM977193	UCSD	GSE50006
GSM563033	MUNICH	GSE13204	GSM977194	UCSD	GSE50006
GSM562889	MUNICH	GSE13204	GSM977195	UCSD	GSE50006
GSM563034	MUNICH	GSE13204	GSM977196	UCSD	GSE50006
GSM563035	MUNICH	GSE13204	GSM977197	UCSD	GSE50006
GSM562891	MUNICH	GSE13204	GSM977198	UCSD	GSE50006
GSM563038	MUNICH	GSE13204	GSM977199	UCSD	GSE50006
GSM563039	MUNICH	GSE13204	GSM977200	UCSD	GSE50006
GSM563040	MUNICH	GSE13204	GSM977201	UCSD	GSE50006
GSM563041	MUNICH	GSE13204	GSM977202	UCSD	GSE50006
GSM563042	MUNICH	GSE13204	GSM977203	UCSD	GSE50006
GSM563043	MUNICH	GSE13204	GSM977204	UCSD	GSE50006

GSM563044	MUNICH	GSE13204	GSM977205	UCSD	GSE50006
GSM562893	MUNICH	GSE13204	GSM977206	UCSD	GSE50006
GSM563045	MUNICH	GSE13204	GSM977207	UCSD	GSE50006
GSM562894	MUNICH	GSE13204	GSM977208	UCSD	GSE50006
GSM563046	MUNICH	GSE13204	GSM977209	UCSD	GSE50006
GSM563047	MUNICH	GSE13204	GSM977210	UCSD	GSE50006
GSM563048	MUNICH	GSE13204	GSM977211	UCSD	GSE50006
GSM563049	MUNICH	GSE13204	GSM977212	UCSD	GSE50006
GSM562896	MUNICH	GSE13204	GSM977213	UCSD	GSE50006
GSM563050	MUNICH	GSE13204	GSM977214	UCSD	GSE50006
GSM562897	MUNICH	GSE13204	GSM977215	UCSD	GSE50006
GSM563051	MUNICH	GSE13204	GSM977216	UCSD	GSE50006
GSM563053	MUNICH	GSE13204	GSM977217	UCSD	GSE50006
GSM563054	MUNICH	GSE13204	GSM977218	UCSD	GSE50006
GSM330930	ROCHE	GSE39671	GSM977219	UCSD	GSE50006
GSM330931	ROCHE	GSE39671	GSM977220	UCSD	GSE50006
GSM330932	ROCHE	GSE39671	GSM977221	UCSD	GSE50006
GSM330933	ROCHE	GSE39671	GSM977222	UCSD	GSE50006
GSM330934	ROCHE	GSE39671	GSM977223	UCSD	GSE50006
GSM330936	ROCHE	GSE39671	GSM977224	UCSD	GSE50006
GSM330938	ROCHE	GSE39671	GSM977225	UCSD	GSE50006
GSM330939	ROCHE	GSE39671	GSM977226	UCSD	GSE50006
GSM330943	ROCHE	GSE39671	GSM977227	UCSD	GSE50006
GSM330944	ROCHE	GSE39671	GSM977228	UCSD	GSE50006
GSM330945	ROCHE	GSE39671	GSM977229	UCSD	GSE50006
GSM330946	ROCHE	GSE39671	GSM977230	UCSD	GSE50006
GSM330947	ROCHE	GSE39671	GSM977231	UCSD	GSE50006
GSM330948	ROCHE	GSE39671	GSM977232	UCSD	GSE50006
GSM330951	ROCHE	GSE39671	GSM977233	UCSD	GSE50006
GSM330952	ROCHE	GSE39671	GSM977234	UCSD	GSE50006
GSM330953	ROCHE	GSE39671	GSM977235	UCSD	GSE50006
GSM330954	ROCHE	GSE39671	GSM977236	UCSD	GSE50006
GSM330955	ROCHE	GSE39671	GSM977237	UCSD	GSE50006
GSM330956	ROCHE	GSE39671	GSM977238	UCSD	GSE50006
GSM330958	ROCHE	GSE39671	GSM977239	UCSD	GSE50006

GSM330959	ROCHE	GSE39671	GSM977240	UCSD	GSE50006
GSM330960	ROCHE	GSE39671	GSM977241	UCSD	GSE50006
GSM330962	ROCHE	GSE39671	GSM977242	UCSD	GSE50006
GSM330964	ROCHE	GSE39671	GSM977243	UCSD	GSE50006
GSM330965	ROCHE	GSE39671	GSM977244	UCSD	GSE50006
GSM330967	ROCHE	GSE39671	GSM977245	UCSD	GSE50006
GSM330968	ROCHE	GSE39671	GSM977246	UCSD	GSE50006
GSM330969	ROCHE	GSE39671	GSM977247	UCSD	GSE50006
GSM330971	ROCHE	GSE39671	GSM977248	UCSD	GSE50006
GSM330972	ROCHE	GSE39671	GSM977249	UCSD	GSE50006
GSM330973	ROCHE	GSE39671	GSM977250	UCSD	GSE50006
GSM330974	ROCHE	GSE39671	GSM977251	UCSD	GSE50006
GSM330975	ROCHE	GSE39671	GSM977252	UCSD	GSE50006
GSM330976	ROCHE	GSE39671	GSM977253	UCSD	GSE50006
GSM330977	ROCHE	GSE39671	GSM977254	UCSD	GSE50006
GSM330978	ROCHE	GSE39671	GSM977255	UCSD	GSE50006
GSM330979	ROCHE	GSE39671	GSM977256	UCSD	GSE50006
GSM330980	ROCHE	GSE39671	GSM977257	UCSD	GSE50006
GSM330981	ROCHE	GSE39671	GSM977258	UCSD	GSE50006
GSM330983	ROCHE	GSE39671	GSM977259	UCSD	GSE50006
GSM330984	ROCHE	GSE39671	GSM977260	UCSD	GSE50006
GSM330987	ROCHE	GSE39671	GSM977261	UCSD	GSE50006
GSM330990	ROCHE	GSE39671	GSM977262	UCSD	GSE50006
GSM330992	ROCHE	GSE39671	GSM977263	UCSD	GSE50006
GSM330994	ROCHE	GSE39671	GSM977264	UCSD	GSE50006
GSM330996	ROCHE	GSE39671	GSM977265	UCSD	GSE50006
GSM330997	ROCHE	GSE39671	GSM977266	UCSD	GSE50006
GSM330998	ROCHE	GSE39671	GSM1690814	IDFCI	GSE10138
GSM330999	ROCHE	GSE39671	GSM1690687	IDFCI	GSE10138
GSM331000	ROCHE	GSE39671	GSM1690688	IDFCI	GSE10138
GSM331001	ROCHE	GSE39671	GSM1690690	IDFCI	GSE10138
GSM331003	ROCHE	GSE39671	GSM1690692	IDFCI	GSE10138
GSM331004	ROCHE	GSE39671	GSM1690693	IDFCI	GSE10138
GSM331005	ROCHE	GSE39671	GSM1690823	IDFCI	GSE10138
GSM331007	ROCHE	GSE39671	GSM1690695	IDFCI	GSE10138

GSM331008	ROCHE	GSE39671	GSM1690912	IDFCI	GSE10138
GSM331009	ROCHE	GSE39671	GSM1690824	IDFCI	GSE10138
GSM331011	ROCHE	GSE39671	GSM1690668	IDFCI	GSE10138
GSM331013	ROCHE	GSE39671	GSM1690826	IDFCI	GSE10138
GSM331014	ROCHE	GSE39671	GSM1690828	IDFCI	GSE10138
GSM331016	ROCHE	GSE39671	GSM1690671	IDFCI	GSE10138
GSM331017	ROCHE	GSE39671	GSM1690697	IDFCI	GSE10138
GSM331019	ROCHE	GSE39671	GSM1690698	IDFCI	GSE10138
GSM331020	ROCHE	GSE39671	GSM1690700	IDFCI	GSE10138
GSM331021	ROCHE	GSE39671	GSM1690831	IDFCI	GSE10138
GSM331022	ROCHE	GSE39671	GSM1690833	IDFCI	GSE10138
GSM331023	ROCHE	GSE39671	GSM1690702	IDFCI	GSE10138
GSM331025	ROCHE	GSE39671	GSM1690703	IDFCI	GSE10138
GSM331026	ROCHE	GSE39671	GSM1690835	IDFCI	GSE10138
GSM331030	ROCHE	GSE39671	GSM1690705	IDFCI	GSE10138
GSM331031	ROCHE	GSE39671	GSM1690707	IDFCI	GSE10138
GSM331033	ROCHE	GSE39671	GSM1690673	IDFCI	GSE10138
GSM331034	ROCHE	GSE39671	GSM1690709	IDFCI	GSE10138
GSM331035	ROCHE	GSE39671	GSM1690710	IDFCI	GSE10138
GSM331037	ROCHE	GSE39671	GSM1690836	IDFCI	GSE10138
GSM331038	ROCHE	GSE39671	GSM1690712	IDFCI	GSE10138
GSM331040	ROCHE	GSE39671	GSM1690713	IDFCI	GSE10138
GSM331041	ROCHE	GSE39671	GSM1690715	IDFCI	GSE10138
GSM331042	ROCHE	GSE39671	GSM1690717	IDFCI	GSE10138
GSM331043	ROCHE	GSE39671	GSM1690840	IDFCI	GSE10138
GSM331044	ROCHE	GSE39671	GSM1690718	IDFCI	GSE10138
GSM331045	ROCHE	GSE39671	GSM1690720	IDFCI	GSE10138
GSM331046	ROCHE	GSE39671	GSM1690914	IDFCI	GSE10138
GSM331047	ROCHE	GSE39671	GSM1690722	IDFCI	GSE10138
GSM331049	ROCHE	GSE39671	GSM1690723	IDFCI	GSE10138
GSM331050	ROCHE	GSE39671	GSM1690841	IDFCI	GSE10138
GSM331052	ROCHE	GSE39671	GSM1690916	IDFCI	GSE10138
GSM331054	ROCHE	GSE39671	GSM1690725	IDFCI	GSE10138
GSM331055	ROCHE	GSE39671	GSM1690917	IDFCI	GSE10138
GSM331056	ROCHE	GSE39671	GSM1690843	IDFCI	GSE10138

GSM331057	ROCHE	GSE39671	GSM1690727	IDFCI	GSE10138
GSM331059	ROCHE	GSE39671	GSM1690674	IDFCI	GSE10138
GSM331060	ROCHE	GSE39671	GSM1690728	IDFCI	GSE10138
GSM331061	ROCHE	GSE39671	GSM1690850	IDFCI	GSE10138
GSM331063	ROCHE	GSE39671	GSM1690851	IDFCI	GSE10138
GSM331065	ROCHE	GSE39671	GSM1690730	IDFCI	GSE10138
GSM331069	ROCHE	GSE39671	GSM1690732	IDFCI	GSE10138
GSM331070	ROCHE	GSE39671	GSM1690735	IDFCI	GSE10138
GSM331071	ROCHE	GSE39671	GSM1690737	IDFCI	GSE10138
GSM331073	ROCHE	GSE39671	GSM1690738	IDFCI	GSE10138
GSM331074	ROCHE	GSE39671	GSM1690860	IDFCI	GSE10138
GSM331075	ROCHE	GSE39671	GSM1690862	IDFCI	GSE10138
GSM331077	ROCHE	GSE39671	GSM1690739	IDFCI	GSE10138
GSM331078	ROCHE	GSE39671	GSM1690741	IDFCI	GSE10138
GSM331081	ROCHE	GSE39671	GSM1690863	IDFCI	GSE10138
GSM331082	ROCHE	GSE39671	GSM1690743	IDFCI	GSE10138
GSM331083	ROCHE	GSE39671	GSM1690745	IDFCI	GSE10138
GSM331084	ROCHE	GSE39671	GSM1690746	IDFCI	GSE10138
GSM331085	ROCHE	GSE39671	GSM1690748	IDFCI	GSE10138
GSM331086	ROCHE	GSE39671	GSM1690865	IDFCI	GSE10138
GSM331087	ROCHE	GSE39671	GSM1690867	IDFCI	GSE10138
GSM331088	ROCHE	GSE39671	GSM1690869	IDFCI	GSE10138
GSM331090	ROCHE	GSE39671	GSM1690870	IDFCI	GSE10138
GSM331091	ROCHE	GSE39671	GSM1690872	IDFCI	GSE10138
GSM331092	ROCHE	GSE39671	GSM1690874	IDFCI	GSE10138
GSM331093	ROCHE	GSE39671	GSM1690876	IDFCI	GSE10138
GSM331096	ROCHE	GSE39671	GSM1690877	IDFCI	GSE10138
GSM331097	ROCHE	GSE39671	GSM1690750	IDFCI	GSE10138
GSM331098	ROCHE	GSE39671	GSM1690752	IDFCI	GSE10138
GSM331099	ROCHE	GSE39671	GSM1690754	IDFCI	GSE10138
GSM331101	ROCHE	GSE39671	GSM1690881	IDFCI	GSE10138
GSM331102	ROCHE	GSE39671	GSM1690755	IDFCI	GSE10138
GSM331104	ROCHE	GSE39671	GSM1690757	IDFCI	GSE10138
GSM331105	ROCHE	GSE39671	GSM1690884	IDFCI	GSE10138
GSM331106	ROCHE	GSE39671	GSM1690759	IDFCI	GSE10138

GSM331107	ROCHE	GSE39671	GSM1690676	IDFCI	GSE10138
GSM331108	ROCHE	GSE39671	GSM1690760	IDFCI	GSE10138
GSM331110	ROCHE	GSE39671	GSM1690762	IDFCI	GSE10138
GSM331111	ROCHE	GSE39671	GSM1690764	IDFCI	GSE10138
GSM331112	ROCHE	GSE39671	GSM1690678	IDFCI	GSE10138
GSM331113	ROCHE	GSE39671	GSM1690766	IDFCI	GSE10138
GSM331115	ROCHE	GSE39671	GSM1690767	IDFCI	GSE10138
GSM331116	ROCHE	GSE39671	GSM1690769	IDFCI	GSE10138
GSM331117	ROCHE	GSE39671	GSM1690771	IDFCI	GSE10138
GSM331118	ROCHE	GSE39671	GSM1690773	IDFCI	GSE10138
GSM331119	ROCHE	GSE39671	GSM1690774	IDFCI	GSE10138
GSM331121	ROCHE	GSE39671	GSM1690885	IDFCI	GSE10138
GSM331122	ROCHE	GSE39671	GSM1690886	IDFCI	GSE10138
GSM331123	ROCHE	GSE39671	GSM1690776	IDFCI	GSE10138
GSM331124	ROCHE	GSE39671	GSM1690778	IDFCI	GSE10138
GSM331125	ROCHE	GSE39671	GSM1690779	IDFCI	GSE10138
GSM331126	ROCHE	GSE39671	GSM1690890	IDFCI	GSE10138
GSM331130	ROCHE	GSE39671	GSM1690781	IDFCI	GSE10138
GSM331131	ROCHE	GSE39671	GSM1690783	IDFCI	GSE10138
GSM331133	ROCHE	GSE39671	GSM1690892	IDFCI	GSE10138
GSM331134	ROCHE	GSE39671	GSM1690784	IDFCI	GSE10138
GSM331135	ROCHE	GSE39671	GSM1690786	IDFCI	GSE10138
GSM331136	ROCHE	GSE39671	GSM1690787	IDFCI	GSE10138
GSM331137	ROCHE	GSE39671	GSM1690681	IDFCI	GSE10138
GSM331138	ROCHE	GSE39671	GSM1690791	IDFCI	GSE10138
GSM331139	ROCHE	GSE39671	GSM1690895	IDFCI	GSE10138
GSM331140	ROCHE	GSE39671	GSM1690792	IDFCI	GSE10138
GSM331144	ROCHE	GSE39671	GSM1690683	IDFCI	GSE10138
GSM331145	ROCHE	GSE39671	GSM1690794	IDFCI	GSE10138
GSM331146	ROCHE	GSE39671	GSM1690898	IDFCI	GSE10138
GSM331147	ROCHE	GSE39671	GSM1690796	IDFCI	GSE10138
GSM331148	ROCHE	GSE39671	GSM1690900	IDFCI	GSE10138
GSM331149	ROCHE	GSE39671	GSM1690902	IDFCI	GSE10138
GSM331151	ROCHE	GSE39671	GSM1690797	IDFCI	GSE10138
GSM331152	ROCHE	GSE39671	GSM1690904	IDFCI	GSE10138

GSM331153	ROCHE	GSE39671	GSM1690905	IDFCI	GSE10138
GSM331155	ROCHE	GSE39671	GSM1690907	IDFCI	GSE10138
GSM331156	ROCHE	GSE39671	GSM1690909	IDFCI	GSE10138
GSM331157	ROCHE	GSE39671	GSM1690799	IDFCI	GSE10138
GSM331158	ROCHE	GSE39671	GSM1690802	IDFCI	GSE10138
GSM331159	ROCHE	GSE39671	GSM1690804	IDFCI	GSE10138
GSM331160	ROCHE	GSE39671	GSM1690806	IDFCI	GSE10138
GSM331161	ROCHE	GSE39671	GSM1690807	IDFCI	GSE10138
GSM331163	ROCHE	GSE39671	GSM1690911	IDFCI	GSE10138
GSM331164	ROCHE	GSE39671	GSM1690810	IDFCI	GSE10138
GSM331166	ROCHE	GSE39671	GSM1690812	IDFCI	GSE10138
GSM331168	ROCHE	GSE39671	GSM1211902	IIDFCI	GSE22762
GSM331169	ROCHE	GSE39671	GSM1211924	IIDFCI	GSE22762
GSM331170	ROCHE	GSE39671	GSM1211947	IIDFCI	GSE22762
GSM331171	ROCHE	GSE39671	GSM1211952	IIDFCI	GSE22762
GSM331172	ROCHE	GSE39671	GSM1211973	IIDFCI	GSE22762
GSM331173	ROCHE	GSE39671	GSM1211975	IIDFCI	GSE22762
GSM331175	ROCHE	GSE39671	GSM1211977	IIDFCI	GSE22762
GSM331176	ROCHE	GSE39671	GSM1211978	IIDFCI	GSE22762
GSM331177	ROCHE	GSE39671	GSM1211979	IIDFCI	GSE22762
GSM331178	ROCHE	GSE39671	GSM1211981	IIDFCI	GSE22762
GSM331179	ROCHE	GSE39671	GSM1211984	IIDFCI	GSE22762
GSM331183	ROCHE	GSE39671	GSM1211990	IIDFCI	GSE22762
GSM331184	ROCHE	GSE39671	GSM1211993	IIDFCI	GSE22762
GSM331185	ROCHE	GSE39671	GSM1211995	IIDFCI	GSE22762
GSM331188	ROCHE	GSE39671	GSM1211999	IIDFCI	GSE22762
GSM331189	ROCHE	GSE39671	GSM1212001	IIDFCI	GSE22762
GSM331192	ROCHE	GSE39671	GSM1212003	IIDFCI	GSE22762
GSM331193	ROCHE	GSE39671	GSM1212004	IIDFCI	GSE22762
GSM331194	ROCHE	GSE39671	GSM1212008	IIDFCI	GSE22762
GSM331195	ROCHE	GSE39671	GSM1212016	IIDFCI	GSE22762
GSM331196	ROCHE	GSE39671	GSM1212017	IIDFCI	GSE22762
GSM331197	ROCHE	GSE39671	GSM1212020	IIDFCI	GSE22762
GSM331201	ROCHE	GSE39671	GSM1212022	IIDFCI	GSE22762
GSM331202	ROCHE	GSE39671	GSM1212030	IIDFCI	GSE22762

GSM331203	ROCHE	GSE39671	GSM1212032	IIDFCI	GSE22762
GSM331207	ROCHE	GSE39671	GSM1212039	IIDFCI	GSE22762
GSM331208	ROCHE	GSE39671	GSM1212044	IIDFCI	GSE22762
GSM331209	ROCHE	GSE39671	GSM1212047	IIDFCI	GSE22762
GSM331210	ROCHE	GSE39671	GSM1212048	IIDFCI	GSE22762
GSM331211	ROCHE	GSE39671	GSM1212049	IIDFCI	GSE22762
GSM331214	ROCHE	GSE39671	GSM1212051	IIDFCI	GSE22762
GSM331215	ROCHE	GSE39671	GSM1212058	IIDFCI	GSE22762
GSM331216	ROCHE	GSE39671	GSM1212061	IIDFCI	GSE22762
GSM331219	ROCHE	GSE39671	GSM1212063	IIDFCI	GSE22762
GSM331222	ROCHE	GSE39671	GSM1212064	IIDFCI	GSE22762
GSM331223	ROCHE	GSE39671	GSM1212065	IIDFCI	GSE22762
GSM331225	ROCHE	GSE39671	GSM1212066	IIDFCI	GSE22762
GSM331226	ROCHE	GSE39671	GSM1212067	IIDFCI	GSE22762
GSM331227	ROCHE	GSE39671	GSM1212069	IIDFCI	GSE22762
GSM331228	ROCHE	GSE39671	GSM1212070	IIDFCI	GSE22762
GSM331229	ROCHE	GSE39671	GSM1212079	IIDFCI	GSE22762
GSM331230	ROCHE	GSE39671	GSM1212080	IIDFCI	GSE22762
GSM331232	ROCHE	GSE39671	GSM1212081	IIDFCI	GSE22762
GSM331233	ROCHE	GSE39671	GSM1212083	IIDFCI	GSE22762
GSM331234	ROCHE	GSE39671	GSM1212087	IIDFCI	GSE22762
GSM331235	ROCHE	GSE39671	GSM1212088	IIDFCI	GSE22762
GSM331236	ROCHE	GSE39671	GSM1212090	IIDFCI	GSE22762
GSM331237	ROCHE	GSE39671	GSM1212096	IIDFCI	GSE22762
GSM331238	ROCHE	GSE39671	GSM1212098	IIDFCI	GSE22762
GSM331240	ROCHE	GSE39671	GSM1212099	IIDFCI	GSE22762
GSM331241	ROCHE	GSE39671	GSM1212102	IIDFCI	GSE22762
GSM331242	ROCHE	GSE39671	GSM1212104	IIDFCI	GSE22762
GSM331243	ROCHE	GSE39671	GSM1212106	IIDFCI	GSE22762
GSM331245	ROCHE	GSE39671	GSM1212110	IIDFCI	GSE22762
GSM331246	ROCHE	GSE39671	GSM1212111	IIDFCI	GSE22762
GSM331248	ROCHE	GSE39671	GSM1212112	IIDFCI	GSE22762
GSM331250	ROCHE	GSE39671	GSM1212113	IIDFCI	GSE22762
GSM331251	ROCHE	GSE39671	GSM1212115	IIDFCI	GSE22762
GSM331252	ROCHE	GSE39671	GSM1212118	IIDFCI	GSE22762

GSM331253	ROCHE	GSE39671	GSM1212120	IIDFCI	GSE22762
GSM331254	ROCHE	GSE39671	GSM1212124	IIDFCI	GSE22762
GSM331257	ROCHE	GSE39671	GSM1212125	IIDFCI	GSE22762
GSM331258	ROCHE	GSE39671	GSM1212126	IIDFCI	GSE22762
GSM331259	ROCHE	GSE39671	GSM1212132	IIDFCI	GSE22762
GSM331260	ROCHE	GSE39671	GSM1212133	IIDFCI	GSE22762
GSM331261	ROCHE	GSE39671	GSM1212134	IIDFCI	GSE22762
GSM331262	ROCHE	GSE39671	GSM1212135	IIDFCI	GSE22762
GSM331266	ROCHE	GSE39671	GSM1212136	IIDFCI	GSE22762
GSM331267	ROCHE	GSE39671	GSM1212138	IIDFCI	GSE22762
GSM331271	ROCHE	GSE39671	GSM1212140	IIDFCI	GSE22762
GSM331272	ROCHE	GSE39671	GSM1212141	IIDFCI	GSE22762
GSM331273	ROCHE	GSE39671	GSM1212146	IIDFCI	GSE22762
GSM331274	ROCHE	GSE39671	GSM1212148	IIDFCI	GSE22762
GSM331275	ROCHE	GSE39671	GSM1212156	IIDFCI	GSE22762
GSM331276	ROCHE	GSE39671	GSM1212157	IIDFCI	GSE22762
GSM331277	ROCHE	GSE39671	GSM1212160	IIDFCI	GSE22762
GSM331279	ROCHE	GSE39671	GSM1212162	IIDFCI	GSE22762
GSM331280	ROCHE	GSE39671	GSM1212164	IIDFCI	GSE22762
GSM331281	ROCHE	GSE39671	GSM1212167	IIDFCI	GSE22762
GSM331282	ROCHE	GSE39671	GSM1212170	IIDFCI	GSE22762
GSM331283	ROCHE	GSE39671	GSM1212171	IIDFCI	GSE22762
GSM331285	ROCHE	GSE39671	GSM1212172	IIDFCI	GSE22762
GSM331286	ROCHE	GSE39671	GSM1212173	IIDFCI	GSE22762

NOTE. Unique GEO accession numbers (GSMxxx), GEO series numbers (GSExxx), and study center are listed for all included samples (n=1,024).
