# Supplementary material for: Subtype assignment of CLL based on B-cell subset associated gene signatures from normal bone marrow – A proof of concept study
Source: PLoS One. 2018 Mar 7;13(3):e0193249. doi: 10.1371/journal.pone.0193249 (PMC5841735; doi:10.1371/journal.pone.0193249)
Supplement: S3 Table — (PDF) [file pone.0193249.s004.pdf]

S3 Table. Characteristics of the CLL Cohorts included in the study.

Cohort	*n*[a]	GSE nr.	Array type	Organization	Age	Gender	Binet Stage	ZAP-70	Cytogenetic Status	IgVH	TTT	Treatment Status	Sample purified before hydridization
**DUKE**	68	GSE 10138	Human Genome U133 plus2	Duke University Medical Center	*NA*	*NA*	*NA*	ZAP-70^{+}: 40 ZAP-70^{-}: 16	No marker/Del13q: 27 Tri12/Del11q: 7 Del17p: 2	mIgVH: 35 uIgVH: 26	*NA*	Untreated	Yes
**IDFCI**	124	GSE 69034	Human Genome U133 plus2	Dana Farber Cancer Institute	*NA*	M: 72	*NA*	ZAP-70^{+}: 42 ZAP-70^{-}: 63	*NA*	mIgVH: 80 uIgVH: 40	*NA*	Untreated	Yes
**IIDFCI**	83	GSE 50006	Human Genome U133 plus2	Dana Farber Cancer Institute	*NA*	M: 47	*NA*	ZAP-70^{+}: 27 ZAP-70^{-}: 52	No marker/Del13q: 49 Tri12/Del11q: 17 Del17p: 6	mIgVH: 44 uIgVH: 37	+	Untreated	Yes
**MUNICH**	127	GSE 22762	Human Genome U133plus2, U133a, and U133b	University Hospital Grosshadern, Ludwig Maximillans-University	62(30-79)	M: 75	A: 56 B-C: 33	*NA*	No marker/Del13q: 89 Tri12/Del11q: 29 Del17p: 8	mIgVH: 61 uIgVH: 66	+	Untreated	PBMC
**PADOVA**	112	GSE 28654	Human Genome U133 plus2	University of Padova	60(37-77)	M: 66	NA	ZAP-70^{+}: 28 ZAP-70^{-}: 61	*NA*	mIgVH: 61 uIgVH: 28	*NA*	Untreated for at least 6 months	Yes
**ROCHE**	318	GSE 13204	Human Genome U133 plus2	Roche Molecular Systems	*NA*	*NA*	*NA*	*NA*	*NA*	*NA*	*NA*	Untreated	Yes
**SAPIENZA**	62	GSE 35935	Human Genome U133 plus2	Sapienza University	69.5(61-84)	M: 45	*NA*	*NA*	*NA*	*NA*	*NA*	Untreated	Leukemic peripheral blood
**UCSD**	130	GSE 39671	Human Genome U133 plus2	University of California, San Diego	*NA*	*NA*	*NA*	*NA*	*NA*	*NA*	+	Untreated	Yes

[a] The patient numbers indicate available, untreated CLL patients, not included in other cohorts. Summary characteristics are shown for cohorts with available data. Age is given as median age in years. NA = not available. Data sets can be accessed online at the Gene Expression Omnibus (http://www.ncbi.nlm.nih.gov/geo/) using the GSE number.
