# Supplementary material for: Subtype assignment of CLL based on B-cell subset associated gene signatures from normal bone marrow – A proof of concept study
Source: PLoS One. 2018 Mar 7;13(3):e0193249. doi: 10.1371/journal.pone.0193249 (PMC5841735; doi:10.1371/journal.pone.0193249)
Supplement: S4 Table — (PDF) [file pone.0193249.s005.pdf]

S4 Table. Agreement between array platforms tested using normal B-cell subsets.

	**Pre-BI**	**Pre-BII**	**Immature**	**Naive**	**Memory**	**Plasma cell**
**Pre-BI**	**3**	0	0	0	0	0
**Pre-BII**	0	**4**	0	0	0	0
**Immature**	0	0	**4**	0	0	0
**Naive**	0	0	0	**10**	0	0
**Memory**	0	0	0	0	**9**	0
**Plasma cell**	0	0	0	0	0	**4**
**Unclassified**	0	0	0	0	0	0

Note: Normal B-cell subsets were harvested from tonsil tissue (accession code GSE56315) and iliac crest (accession code GSE107843) from adult donors after written consent and hybridized to Human Genome Affymetrix U133 plus 2.0 arrays. The BAGS classifier generated using the Human Exon 1.0 ST Array platform was able to classify all B-cell subsets with 100% accuracy.
