# Supplementary material for: Subtype assignment of CLL based on B-cell subset associated gene signatures from normal bone marrow – A proof of concept study
Source: PLoS One. 2018 Mar 7;13(3):e0193249. doi: 10.1371/journal.pone.0193249 (PMC5841735; doi:10.1371/journal.pone.0193249)
Supplement: S5 Table — (PDF) [file pone.0193249.s006.pdf]

S5 Table. BAGS classifier genes

		BAGS Subtypes					
**ENSEMBL Gene ID[a]**	**HGNC Symbol**	**Pre-BI**	**Pre-BII**	**Immature**	**Naive**	**Memory**	**Plasma cell**
ENSG00000004468	CD38	0	0	0,055148822	0	-0,195897288	0
ENSG00000005187	ACSM3	-0,079835662	0,383419171	0	0	0	0
ENSG00000007062	PROM1	0,006368928	0	0	0	0	0
ENSG00000012124	CD22	0	0	0	0	0	-0,009728154
ENSG00000023445	BIRC3	-0,095499243	0	0	0	0	0
ENSG00000026751	SLAMF7	0	0	0	0	0	0,081333674
ENSG00000042980	ADAM28	0	-0,120277562	0	0,12739731	0	0
ENSG00000048462	TNFRSF17	-0,174281415	0,135951007	-0,163019931	0	0	0,121841186
ENSG00000051108	HERPUD1	0	0	0	0	0	0,016813158
ENSG00000051180	RAD51	0	0,015917164	0	0	0	0
ENSG00000057657	PRDM1	0	0	0	0	0	0,066261657
ENSG00000060138	YBX3	0	0	0	0	-0,03089936	0
ENSG00000070540	WIPI1	0	0	0	0	0	0,036604349
ENSG00000071537	SEL1L	0	0	0	0	0	0,057865137
ENSG00000071575	TRIB2	0	0	0,006207067	0	0	0
ENSG00000075420	FNDC3B	0	0	0	0	0	0,051806894
ENSG00000076003	MCM6	0	0	-0,014482045	0	0	0
ENSG00000077238	IL4R	0	0	0	0,435041027	-0,186048666	0
ENSG00000077943	ITGA8	0	0	0	0	0	0,07554695
ENSG00000078589	P2RY10	-0,007941132	0	0	0	0	0
ENSG00000079263	SP140	0	-0,065700556	0,059031616	0	0	0
ENSG00000082293	COL19A1	0	0	0	0,047623575	0	0
ENSG00000086967	MYBPC2	0	0	0,030470208	0	0	0
ENSG00000087086	FTL	0	-0,001774317	0	0	0	0
ENSG00000090376	IRAK3	0	0	0	0,025492407	0	0
ENSG00000091409	ITGA6	0	0	0	0	0	0,085961417
ENSG00000091972	CD200	0	0	0	0,219445183	-0,053181588	0
ENSG00000092853	CLSPN	0	0,059381831	0	0	0	0
ENSG00000093009	CDC45	0	0,021236058	0	0	0	0
ENSG00000094804	CDC6	0	9,43522E-06	0	0	0	0
ENSG00000095015	MAP3K1	-0,037929964	0	0	0	0	0
ENSG00000096006	CRISP3	0	0	0	0	0,007467653	0
ENSG00000096696	DSP	0	0	0	0,008633427	0	0
ENSG00000100219	XBP1	0	0	0	0	0	0,022246552
ENSG00000100721	TCL1A	-0,252421595	0,215064951	0,068462648	0,254363422	-0,342169495	0
ENSG00000101336	HCK	0	0	0,050612299	-0,011804743	0	0
ENSG00000102218	RP2	0	0	0	-3,26893E-05	0	0
ENSG00000102445	KIAA0226L	0	0	0	0	0	-0,06030621
ENSG00000102580	DNAJC3	0	0	0	0	0	0,048508443
ENSG00000103528	SYT17	0	0	0	0,291140971	0	0
ENSG00000104432	IL7	0	0	0	0	0,15202983	0
ENSG00000104921	FCER2	0	0	0	0,081703434	0	0
ENSG00000105694	TCEB1P28	0	0	0	0,015525288	0	0
ENSG00000105854	PON2	0	0	0,008386052	0	0	0
ENSG00000105889	STEAP1B	0	0	0	0,059666484	0	0
ENSG00000105967	TFEC	0	0	0	-0,026270125	0,272774074	0
ENSG00000106624	AEBP1	0	0	0,008085875	0	0	0
ENSG00000107447	DNTT	0,238519145	0	0	0	0	0
ENSG00000109674	NEIL3	0	0,004239307	0	0	0	0
ENSG00000109787	KLF3	0	0	0	0,001043312	0	0

ENSG00000109805	NCAPG	0	0,01656183	-0,032895735	0	0	0
ENSG00000110077	MS4A6A	0	0	0,208106157	0	0	0
ENSG00000111913	FAM65B	0	0	0	0	0,030021104	-0,009183778
ENSG00000112290	WASF1	0	0,211256246	0,163415154	-0,108316254	0	0
ENSG00000113615	SEC24A	0	0	0	0	0	0,025387114
ENSG00000114346	ECT2	0	0,002962178	0	0	0	0
ENSG00000114948	ADAM23	-0,045871791	0,136902635	0,271397044	-0,112268779	0	0
ENSG00000115956	PLEK	0	0	0	0,046113831	0	0
ENSG00000115993	TRAK2	0	0	0	0,068802022	0	0
ENSG00000116748	AMPD1	0	0	0	0	0	0,215735065
ENSG00000116815	CD58	0	0	-0,032324773	0	0	0
ENSG00000117090	SLAMF1	0	0	0	0	-0,013233152	0
ENSG00000118113	MMP8	0	0	0	0	0,08597789	0
ENSG00000118193	KIF14	0	0,002393982	0	0	0	0
ENSG00000118985	ELL2	0	0	0	0	0	0,032305437
ENSG00000119782	FKBP1B	0	0	0	0	0,057395872	0
ENSG00000120049	KCNIP2	0	0	0	0,095575932	0	0
ENSG00000121152	NCAPH	0	0,02045612	0	0	0	0
ENSG00000121807	CCR2	0	0	0	0	0	0,021441048
ENSG00000121895	TMEM156	0	-0,043472633	0	0	0	0
ENSG00000122025	FLT3	0,104734484	0	0	0	0	0
ENSG00000122862	SRGN	0	0	0,003228003	-0,043881598	0	0
ENSG00000123352	SPATS2	0	0	0	0	0	0,011213308
ENSG00000123975	CKS2	0	0,039191105	0,0735212	-0,32861928	0	0
ENSG00000124942	AHNAK	0	-0,064162651	0	0	0,030115394	0
ENSG00000126353	CCR7	0	0	0	0,184076656	0	0
ENSG00000128641	MYO1B	0	0	0	0,000895254	0	0
ENSG00000128833	MYO5C	0,124529232	0	0	0	0	0
ENSG00000129173	E2F8	0	0,117496115	0	0	0	0
ENSG00000129824	RPS4Y1	0	0	0	0,050504228	0	0
ENSG00000131002	TXLNGY	0	-0,012078363	0	0	0	0
ENSG00000131724	IL13RA1	0	0	0	0,14567796	0	0
ENSG00000131781	FMO5	0	0	0	0,016333712	0	0
ENSG00000132274	TRIM22	0	0	0	0	0	-0,015002068
ENSG00000133119	RFC3	0	0,004903487	0	0	0	0
ENSG00000133935	C14orf1	0	0	-0,034070368	0	0	0
ENSG00000134061	CD180	-0,121919412	0	0	0	0	0
ENSG00000134460	IL2RA	0	0	0	0	0,146896989	0
ENSG00000135048	TMEM2	0	0	0	0,0168304	0	0
ENSG00000135116	HRK	0	0	0,062288047	0	0	0
ENSG00000135318	NT5E	0	0	0	0,145090155	0	0
ENSG00000136982	DSCC1	0	0,000790732	0	0	0	0
ENSG00000137101	CD72	0	0	0	0	0	-0,010197401
ENSG00000137265	IRF4	-0,037015874	0,114326553	0	0	0	0
ENSG00000138180	CEP55	0	0,00775363	0	0	0	0
ENSG00000138587	MNS1	0	0,099680726	0	0	0	0
ENSG00000138639	ARHGAP24	0	0	0	0	0,084370548	0
ENSG00000138778	CENPE	0	0,028131749	0	0	0	0
ENSG00000141293	SKAP1	0	0	0	0,082320327	0	0
ENSG00000142731	PLK4	0	0,021804705	0	0	0	0
ENSG00000143476	DTL	0	0,059506275	0	0	0	0
ENSG00000143756	FBXO28	0	0	0	0,051248175	0	0
ENSG00000145386	CCNA2	0	0,00227826	0	0	0	0
ENSG00000147168	IL2RG	0	-0,092324224	0	0	0	0
ENSG00000149054	ZNF215	0	0	0	0	0	0,050655318

ENSG00000149177	PTPRJ	0	0	0,13923948	0	0	0
ENSG00000152253	SPC25	0	0,096086846	0	0	0	0
ENSG00000152256	PDK1	0	0	0	0	0	0,051758699
ENSG00000152689	RASGRP3	0	0	0	0,066087802	0	0
ENSG00000152785	BMP3	0	0	0,210912983	-0,021609218	0	0
ENSG00000153064	BANK1	0	0	0	0	0,053426533	0
ENSG00000153253	SCN3A	0	0	0	0,013136137	0	0
ENSG00000156136	DCK	0	0	0	0	0	-0,096811026
ENSG00000156738	MS4A1	-0,09674709	-0,035599607	0,203383245	0	0,183879563	-0,125120737
ENSG00000157554	ERG	0,080812772	0	0	0	0	0
ENSG00000158164	TMSB15A	0	0,161372502	0	0	0	0
ENSG00000158481	CD1C	0	0	0	0	0,220838254	0
ENSG00000160683	CXCR5	0	0	0	0,024110902	0	0
ENSG00000161405	IKZF3	-0,31501003	0,005680736	0	0	0	0
ENSG00000162188	GNG3	0	-0,019196563	0,233395141	0	0	0
ENSG00000162692	VCAM1	0,00650845	0	0	0	0	0
ENSG00000162924	REL	0	0	0	0	0	-0,052314869
ENSG00000163106	HPGDS	0,033544742	0	0	0	0	0
ENSG00000163563	MNDA	0	0	0	0	0,040735911	0
ENSG00000163564	PYHIN1	0	0	0	0	0,0098032	0
ENSG00000163568	AIM2	0	0	-0,028023942	0	0,441238531	0
ENSG00000163808	KIF15	0	0,022153672	0	0	0	0
ENSG00000164543	STK17A	0	0	0	0,01605094	0	0
ENSG00000164821	DEFA4	0	0	0,062887765	0	0	0
ENSG00000166086	JAM3	0	0	0	0	0,007661801	0
ENSG00000168078	PBK	0	0,004008476	0	0	0	0
ENSG00000169397	RNASE3	-0,044264185	0	-0,022762663	0	0	0
ENSG00000169413	RNASE6	0	-0,173432968	0,089903968	0	0	0
ENSG00000169508	GPR183	0	0	0	0	0,207631154	0
ENSG00000169575	VPREB1	0	0,005245374	0	-0,02397329	0	0
ENSG00000171848	RRM2	0	0,11862101	0	0	0	0
ENSG00000173141	MRPL57	0	0	-0,01772027	0	0	0
ENSG00000173208	ABCD2	0	0	0	-0,055545843	0	0
ENSG00000173559	NABP1	0	0	0	0	0,017519542	0
ENSG00000174059	CD34	0,176848326	-0,053565491	0	0	0	0
ENSG00000174371	EXO1	0	0,042747304	0	0	0	0
ENSG00000174944	P2RY14	0	0	0	0,31531888	-0,213049831	0
ENSG00000177575	CD163	0,013386319	0	0	0	0	0
ENSG00000179344	HLA-DQB1	-0,0157371	0	0	0	0	0
ENSG00000181631	P2RY13	0	0	0	0	0,02862413	0
ENSG00000181690	PLAG1	0	0	-0,019735914	0	0	0
ENSG00000184226	PCDH9	0	0,007525214	0,199798101	0	0	0
ENSG00000184566		0	0	0	-0,017447206	0	0
ENSG00000188404	SELL	0	0	0	0,010491759	0	0
ENSG00000196368	NUDT11	0,041081648	0	0	0	0	0
ENSG00000196549	MME	0	0	0	-0,022613468	0	0
ENSG00000196584	XRCC2	0	0,025030886	0	0	0	0
ENSG00000197429	IPP	-0,012224959	0	0	0	0	0
ENSG00000197879	MYO1C	0	0	0,030171869	0	0	0
ENSG00000198692	EIF1AY	0	0	-0,04238152	0,151798094	0	0
ENSG00000198825	INPP5F	0	0	-0,045977305	0,045464168	0	0
ENSG00000204287	HLA-DRA	0	0	0	0	0,01761542	-0,240799272
ENSG00000204389	HSPA1A	0	-0,083658749	0	0	0	0
ENSG00000204852	TCTN1	0	0	0	0,056953717	0	0
ENSG00000211598	IGKV4-1	0	0	0	0	0	0,051039937

ENSG00000211640	IGLV6-57	0	0	0	0	0	0,10268902
ENSG00000211653	IGLV1-40	0	0	0,034170107	0	0	0,072178525
ENSG00000213005	PTTG3P	0	0	0,071672376	0	0	0
ENSG00000218690	HIST1H2APS4	0	0,018873909	0	0	0	0
ENSG00000220506		0	0	-0,02943808	0,138309229	0	0
ENSG00000220541		0	0	0,048105374	-0,006145234	0	0
ENSG00000227234	SPANXB1	0,144284596	0	0	0	0	0
ENSG00000231991	ANXA2P2	-0,017051882	0	0	-0,072334697	0	0
ENSG00000241106	HLA-DOB	-0,243722552	0	0	0	0	0
ENSG00000242574	HLA-DMB	0	0	0	0	0	-0,146703441
ENSG00000254087	LYN	-0,014003892	0	0	0	0	0
ENSG00000261371	PECAM1	0	-0,14321088	0	0	0	0
ENSG00000263639	MSMB	0,042322611	0	0	0	0	0
ENSG00000265241	RBM8A	0	0	0,077893246	0	0	0
ENSG00000269404	SPIB	-0,104827171	0	0,121865529	0	0	0
ENSG00000273703	HIST1H2BM	0	0,057518809	0	0	0	0
ENSG00000275302	CCL4	0	0	0,318426424	0	0	0
ENSG00000277734	TRAC	0	0	0,184305101	0	0	0
ENSG00000277775	HIST1H3F	-0,063830715	0,253385852	-0,021812031	0	0	0
ENSG00000278272	HIST1H3C	0	0,099113231	0	0	0	0
ENSG00000278463	HIST1H2AB	0	0,110638332	0	0	0	0
ENSG00000278588	HIST1H2BI	0	0,146629096	-0,036035928	0,071288536	0	0
ENSG00000278705	HIST1H4B	0	0	-0,062725095	0	0	0
ENSG00000279530		0	0	0,02168593	0	0	0

[a]Ensembl gene id and corresponding HGNC symbols for classifier genes (*n*=184), derived from sternal bone marrow B-cell data, are listed along with relative expression values for the respective classifier-specific subtype(s).
