# Supplementary material for: Subtype assignment of CLL based on B-cell subset associated gene signatures from normal bone marrow – A proof of concept study
Source: PLoS One. 2018 Mar 7;13(3):e0193249. doi: 10.1371/journal.pone.0193249 (PMC5841735; doi:10.1371/journal.pone.0193249)
Supplement: S6 Table — (PDF) [file pone.0193249.s007.pdf]

S6 Table. BAGS assignment, clinical variables, and outcome

	1	2	3
	**Hazard ratio (95 % CI) *P***	**Hazard ratio (95 % CI) *P***	**Hazard ratio (95 % CI) *P***
**Gender**			
Female			1
Male			1.033 (0.643 to 1.659)<br>p < 0.895
**BAGS subtype**			
Memory	1	1	1
Pre-BI	1.562 (0.826 to 2.951)<br>p = 0.170	2.230 (1.166 to 4.264)<br>**p = 0.015***	1.709 (0.877 to 3.331))<br>p = 0.115
Pre-BII	2.732 (0.776 to 9.616)<br>p = 0.118	8.836 (2.507 to 31.501)<br>**p = 0.001***	4.210 (1.150 to 15.409)<br>**p = 0.030***
Immature	1.458 (0.662 to 3.212))<br>p = 0.349	1.098 (0.426 to 2.829)<br>p = 0.846	1.412 (0.562 to 3.544)<br>p = 0.463
Naive	1.392 (0.725 to 2.671)<br>p = 0.320	1.786 (0.921 to 3.464)<br>p = 0.086	1.636 (0.827 to 3.237)<br>p = 0.158
Plasma cell	1.170 (0.405 to 3.378)<br>p = 0.772	1.023 (0.353 to 2.966)<br>p = 0.967	1.345 (0.453 to 3.988)<br>p = 0.594
Unclassified	0.807 (0.415 to 1.568)<br>p = 0.527	1.335 (0.663 to 2.692)<br>p = 0.419	1.041 (0.506 to 2.140)<br>p = 0.913
**IgVH status**			
uIgVH	1		1
mIgVH	0.222 (0.137 to 0.359)<br>**p < 0.001***		0.280 (0.166 to 0.469)<br>**p < 0.001***
**Cytogenetic status**			
No marker/13q		1	1
Tri12/11q		3.173 (1.905 to 5.284)<br>**p < 0.001***	2.103 (1.253 to 3.527)<br>**p = 0.005***
Del17p		2.702 (1.159 to 6.295)<br>**p = 0.021***	2.150 (0.907 to 5.094)<br>p = 0.082
Cohort			
IIDFCI			1
Munich			3.001 (1.739 to 5.180)<br>**< 0.001***
**Observations**	164	155	154

Note: BAGS assignments for individual BAGS subtypes and clinical variables were associated to outcome (time to treatment) using multivariate Cox proportional hazards regression analysis (7.5 events per variable). The Munich and IIDFCI cohorts were used. Hazards ratios are shown in the stepwise multivariate model: 1) BAGS subtypes and IgVH status, observations N = 164. 2) BAGS subtypes and cytogenetic status, observations N = 155. 3) All variables (gender, BAGS subtypes, IgVH, cytogenetic status), observations N = 154. The Munich cohort and IIDFCI cohorts was used.
