# Supplementary material for: Subtype assignment of CLL based on B-cell subset associated gene signatures from normal bone marrow – A proof of concept study
Source: PLoS One. 2018 Mar 7;13(3):e0193249. doi: 10.1371/journal.pone.0193249 (PMC5841735; doi:10.1371/journal.pone.0193249)

S1 Fig. Time to first treatment in the Munich, UCSD, and IIDFCI cohorts. Cumulative incidence curves show years elapsed from the time of diagnostic GEP until the commencement of initial treatment.

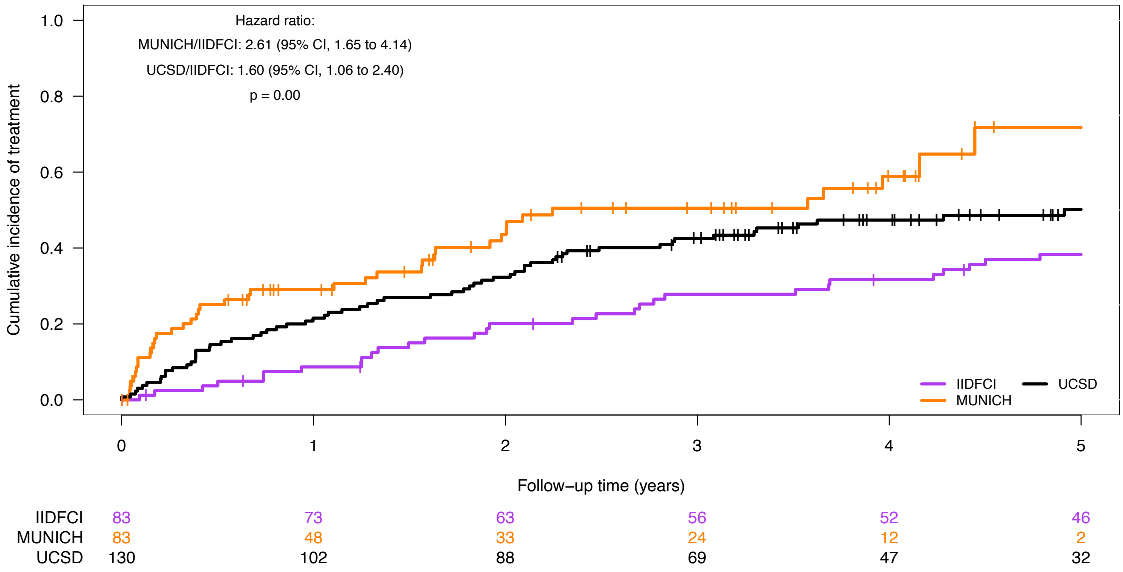

Supplement: S1 Fig — (PDF) [file pone.0193249.s008.pdf]
