# Supplementary figures and images for: Subtype assignment of CLL based on B-cell subset associated gene signatures from normal bone marrow – A proof of concept study
Source: PLoS One. 2018 Mar 7;13(3):e0193249. doi: 10.1371/journal.pone.0193249 (PMC5841735; doi:10.1371/journal.pone.0193249)

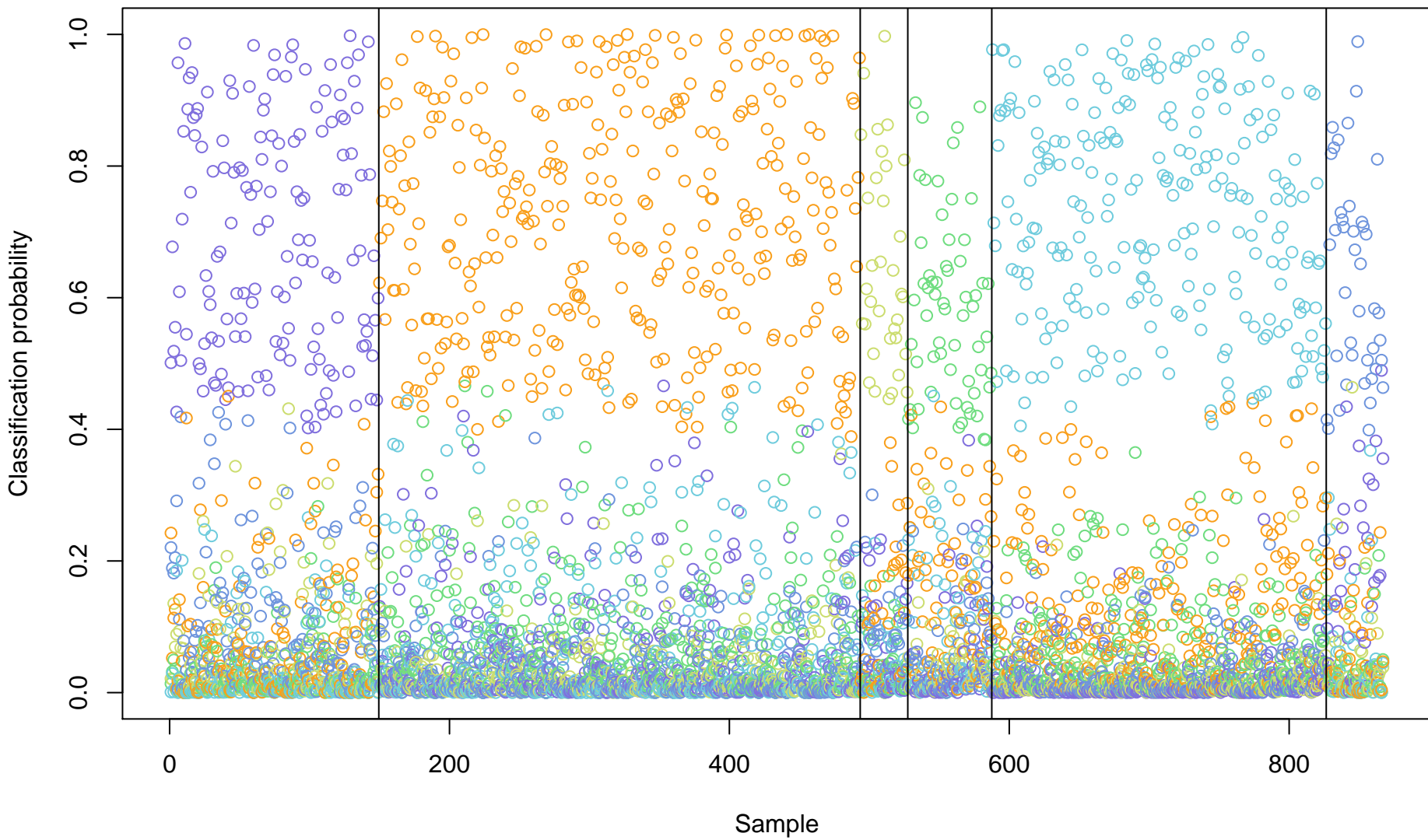

Pre-BI Pre-BII Immature Naive Memory Plasmacell

Supplement: S2 Fig — (PDF) [file pone.0193249.s009.pdf]

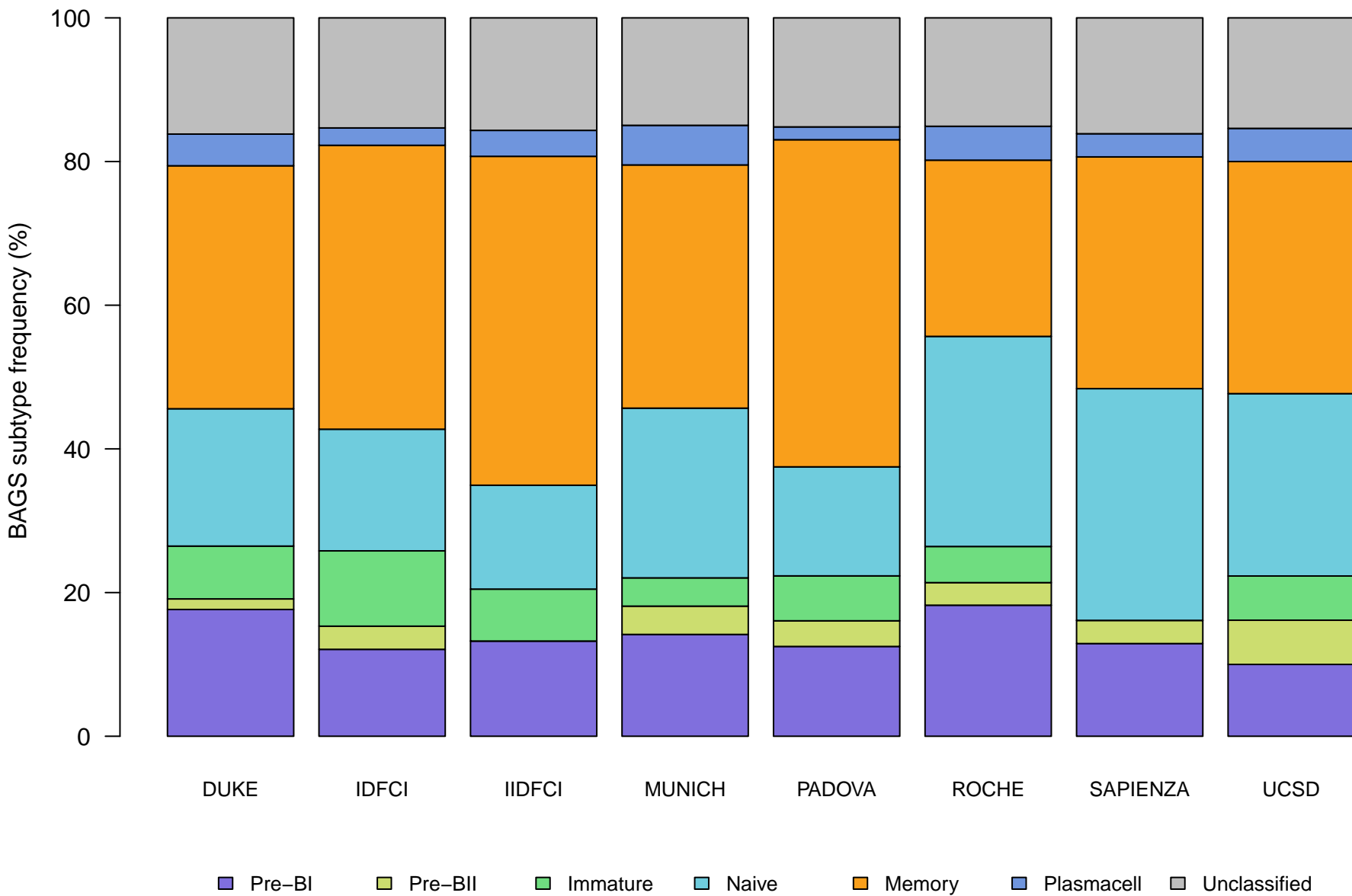

Supplement: S3 Fig — (PDF) [file pone.0193249.s010.pdf]
