# Supplementary material for: Subtype assignment of CLL based on B-cell subset associated gene signatures from normal bone marrow – A proof of concept study
Source: PLoS One. 2018 Mar 7;13(3):e0193249. doi: 10.1371/journal.pone.0193249 (PMC5841735; doi:10.1371/journal.pone.0193249)

S4 Fig. Association between IgVH mutation status and BAGS subtype. For each subtype the proportion of samples with mutated and unmutated IgVH are shown. **(a)** All subtypes, **(b)** All subtypes divided into early, naïve and late subtypes. The Munich, Duke, Padova, and IDFCI cohorts were used (n = 337). Unclassified samples were not included. Tests for significantly different distributions between groups were calculated using Fisher's exact test (**(a)** P = < .001 and **(b)**: P = < .001, early vs. late; P = < .001, naïve vs. late; P = .01).

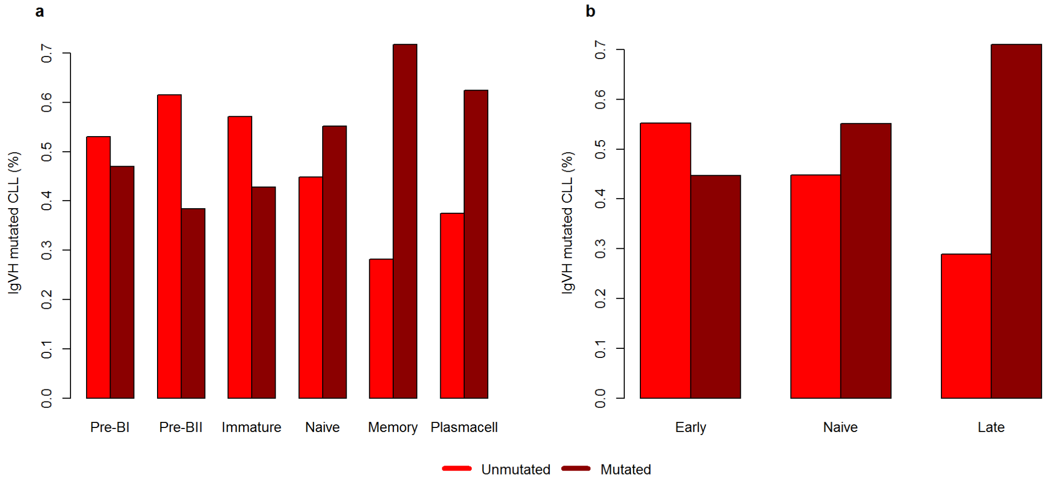

Supplement: S4 Fig — (PDF) [file pone.0193249.s011.pdf]
