# Supplementary material for: Subtype assignment of CLL based on B-cell subset associated gene signatures from normal bone marrow – A proof of concept study
Source: PLoS One. 2018 Mar 7;13(3):e0193249. doi: 10.1371/journal.pone.0193249 (PMC5841735; doi:10.1371/journal.pone.0193249)

S5 Fig. Analyses of the prognostic impact of subtyping according to BAGS on TTT in watch-and-wait CLL. The cumulative incidence of treatment. (a) All subtypes. (b) All subtypes divided as early (pre-), naïve, and late (post-germinal). Only data from the IIDFCI, and UCSD cohort were used (n = 180), as the Munich cohort was excluded.

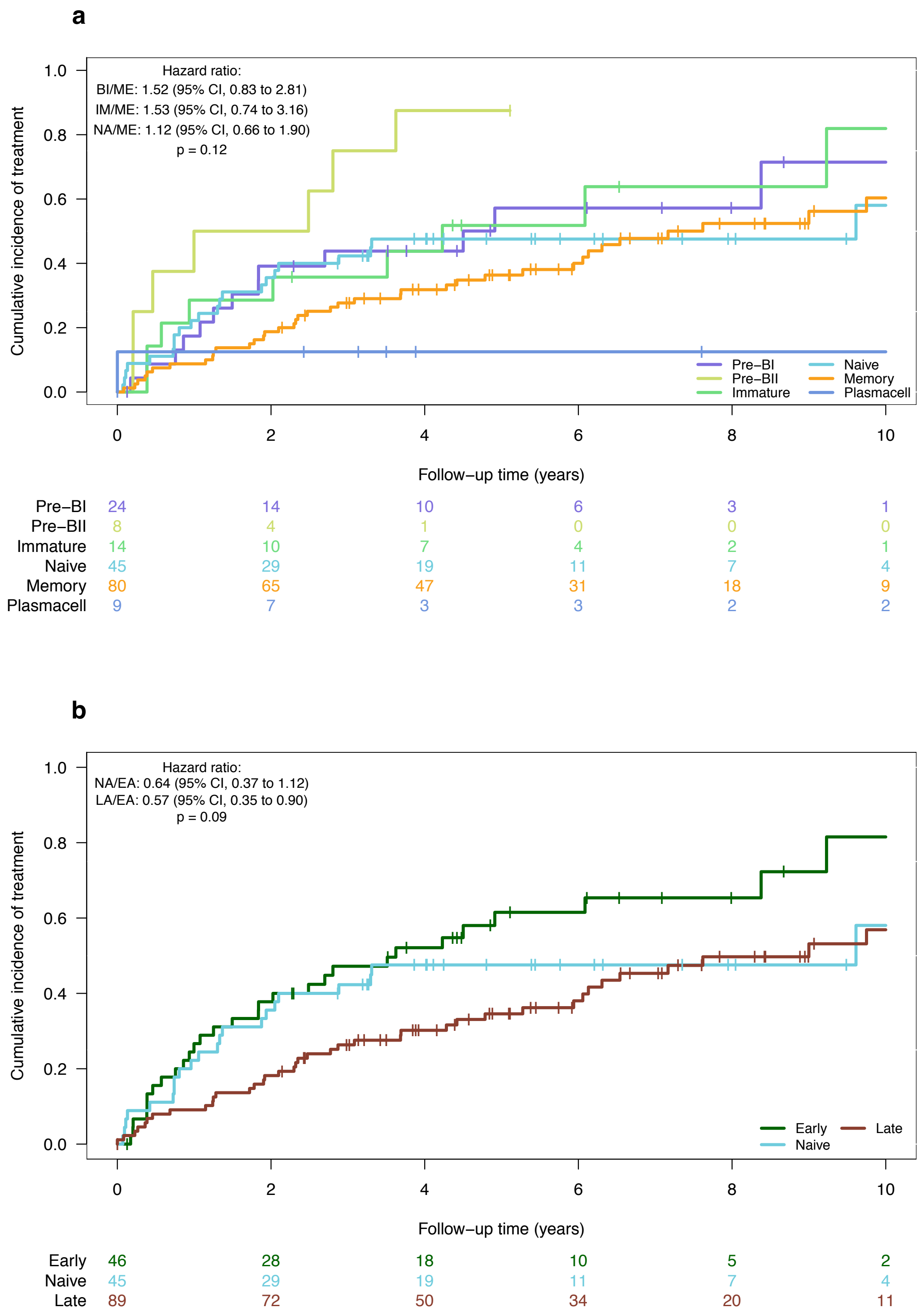

Supplement: S5 Fig — (PDF) [file pone.0193249.s012.pdf]
